# Beneficial behavioral effects of chronic cerebral dopamine neurotrophic factor (CDNF) infusion in the N171-82Q transgenic model of Huntington’s disease

**DOI:** 10.1038/s41598-023-28798-4

**Published:** 2023-02-20

**Authors:** P. Stepanova, D. Kumar, K. Cavonius, J. Korpikoski, J. Sirjala, D. Lindholm, M. H. Voutilainen

**Affiliations:** 1 Faculty of Pharmacy, University of Helsinki, Helsinki, Finland; 2 Biomedicum, Aiforia Technologies, Helsinki, Finland; 3 Medicum, Department of Biochemistry and Developmental Biology, Faculty of Medicine, University of Helsinki, Helsinki, Finland; 4 Minerva Foundation Institute for Medical Research, Biomedicum, University of Helsinki, Helsinki, Finland

**Keywords:** Huntington's disease, Movement disorders, Neurodegenerative diseases, Regenerative medicine

## Abstract

Huntington’s disease (HD) is a progressive inherited neurological disease characterized by the degeneration of basal ganglia and the accumulation of mutant huntingtin (mHtt) aggregates in specific brain areas. Currently, there is no treatment for halting the progression of HD. Cerebral dopamine neurotrophic factor (CDNF) is a novel endoplasmic reticulum located protein with neurotrophic factor properties that protects and restores dopamine neurons in rodent and non-human primate models of Parkinson’s disease. Our recent study showed that CDNF improves motor coordination and protects NeuN positive cells in a Quinolinic acid toxin rat model of HD. Here we have investigated the effect of chronic intrastriatal CDNF administration on behavior and mHtt aggregates in the N171-82Q mouse model of HD. Data showed that CDNF did not significantly decrease the number of mHtt aggregates in most brain regions studied. Notably, CDNF significantly delayed the onset of symptoms and improved motor coordination in N171-82Q mice. Furthermore, CDNF increased *BDNF* mRNA level in hippocampus in vivo in the N171-82Q model and BDNF protein level in cultured striatal neurons. Collectively our results indicate that CDNF might be a potential drug candidate for the treatment of HD.

## Introduction

Huntington’s disease (HD) is an inherited neurodegenerative disease in which striatal and cortical neurons degenerate^1,2^. The cause of HD is the expression of mutant huntingtin (mHtt) with polyQ repeat expansion present in the N-terminal part of the protein^3^. Mouse models expressing N-terminal mHtt show the formation of neuronal intranuclear inclusions within the striatum and cortex^4,5^ corresponding to the pathology observed in HD patients^6,7^, including motor deficits and cognitive impairments.

In this study, we used the genetic N171-82Q mouse model of HD, which has a longer lifespan than the R6/2 transgenic model but a similar phenotype^8–10^. In this model, cDNA encoding a 171-amino acid N-terminal fragment of Htt containing 82 glutamine repeats is expressed under the mouse prion protein promoter^11^, which drives the expression primarily in neurons^12^. N171-82Q mice develop behavioral changes including clasping of hindlimbs, tremor and weight loss from 8 to 12 weeks of age and with an endpoint at 16–22 weeks^13–15^. N171-82Q mice have been used in preclinical trials for screening therapeutic drug candidates as they show fast symptom progression and demonstrates the wide expression of mHtt inclusions in the brain^9^. Especially, there are intranuclear and cytoplasmic perinuclear mHtt inclusions in the brains of N171-82Q mice^12^. The function of mHtt inclusions is still largely unclear, but data suggest that N-terminal mHtt is present in a larger complex with other proteins in the brain^16^.

Currently, there is no effective treatment to stop neurodegeneration in the affected brain areas of HD patients. Neurotrophic factors (NTFs) are secretory proteins that are involved in the survival, development, differentiation and functional recovery of neurons. There are decreased levels of NTFs in the brains of patients with neurodegenerative diseases including Parkinson´s disease (PD), Alzheimer´s disease (AD) and HD^17–19^, and these factors have therefore also been tested in preclinical and clinical trials of Amyotrophic lateral sclerosis (ALS), AD, PD and HD^20–24^.

Cerebral dopamine neurotrophic factor (CDNF) is a conserved secreted protein that is expressed in neuronal and non-neuronal tissue^25–28^. CDNF is a member of the mesencephalic astrocyte-derived neurotrophic factor (MANF)-CDNF family, which differs from classical NTFs in terms of structure, biological functions and diffusion capacity^29^. CDNF contains two structural domains: the N-terminal domain, which most likely binds with lipids, and the C-terminal domain, which contains the KTEL endoplasmic reticulum (ER) retention signal^30,31^. CDNF is located and secreted in the lumen of the ER^21,32^. Previous data on the effects of CDNF on ER stress markers support the hypothesis of direct regulation of UPR pathways by CDNF^22,33^. Moreover, recent data showed that CDNF interacts with the main ER chaperone Bip (as known as GRP78)^34^, is upregulated and secreted in response to experimental ER stress, and protects cells from ER stress-induced cell death both in vitro and in vivo^24,35^.

The diffusion of CDNF in brain is more prolonged and distributed widely than that of other NTFs, and the half-life is approximately 5.5 h^23,36,37^. Previous studies have demonstrated that CDNF exerts neuroprotective and neurorestorative effects in rodent and non-human primate models of PD^27,37–39^. Notably, in the phase I/II clinical trials on PD patients, CDNF demonstrated safety and showed increased dopamine transporter availability in the putamen of some patients in positron emission computer tomography (DAT PET) analyses^21^.

In our previous study, we demonstrated that a single intrastriatal injection of CDNF has a positive effect in the quinolinic acid (QA)-toxin model of HD^23^. CDNF improved motor behavior and increased the NeuN- and doublecortin-immunopositive cell populations in vivo and protected striatal neurons after QA toxin-induced cell death in vitro. These findings highlighted the importance of CDNF as a potential drug candidate also in HD. However, studying the effect of CDNF further in vivo using genetic models of HD is crucial for its possible future application in clinical trials.

In the present study, we infused CDNF for 4 weeks unilaterally into the striatum of the N171-82Q mice using an Alzet minipump to study the effect of CDNF on motor coordination. We were additionally interested in studying the effect of CDNF on mHtt aggregates in these mice using a deep learning neural network method developed for this purpose.

Our results demonstrate that CDNF alleviated motor dysfunction in N171-82Q mice by decreasing the percentage of animals with clasped hindlimbs. Additionally, CDNF improved motor coordination in N171-82Q females as measured by rotarod and the beam walking tests. Using a deep learning method, we concluded that CDNF has a tendency for reducing the EM48 immunoreactivity and the number of intranuclear aggregates in the striatum of female mice after CDNF treatment. CDNF further increased the mRNA level of *BDNF* in the brains of N171-82Q mice, as well as BDNF protein levels in neuronal cultures. The results also suggest that CDNF can affect ER stress at the high expression injection site, with a tendency to reduce ER stress markers in the striatum following CDNF administration. The results support that CDNF significantly improves behavior in the N171-82Q mouse model of HD that may be beneficial for future therapies and as such warrants further investigations.

## Materials and methods

### Animals

Adult female and male C57BL/6JRccHsd × B6C3-Tg (HD82Gln)81Gschi/J mice (9 weeks) were used for the chronic CDNF infusion experiment. The wild-type (WT) naïve animals (C57BL/6JRccHsd) were used as a control group. Animal experiments were approved by the Finnish National Board of Animal Experiments and were carried out according to the European Community guidelines for the use of experimental animals with the license numbers, ESAVI/8897/04.10.07/2017 and ESAVI/27113/2020. Reporting in the manuscript follows the recommendations in the ARRIVE guidelines.

### Chronic CDNF delivery

**Figure 1:**
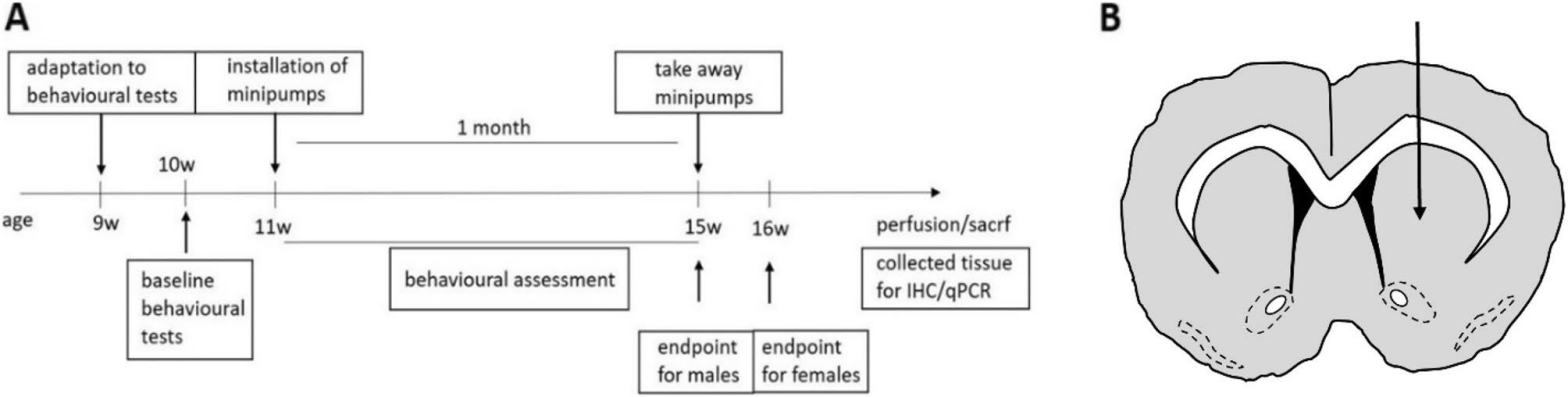
Study design. (**A**) Experimental timeline. An intrastriatally implanted cannula connected to a minipump releasing vehicle (PBS) or CDNF was installed subcutaneously at 11 weeks of age of N171-82Q mice. Minipumps were removed at 15 weeks. Behavioral tests were conducted twice per week. Animals were sacrificed for collecting tissues at 15 weeks of age for N171-82Q male mice and 16 weeks for N1721-82Q female mice. (**B**) Scheme of cannula placement (A/P + 0.86; M/L − 1.8; D/V − 3.0).

Mice were anaesthetised under isoflurane anaesthesia (3.5% during induction and 2.5–3% during maintenance) and placed in a stereotaxic apparatus. A tip of an infusion cannula was implanted into the right striatum (A/P + 0.86; M/L − 1.8; D/V − 3.0), connected via a plastic catheter tube to an Alzet minipump (model 1004, Agnthos, Sweden). The Alzet minipump was implanted subcutaneously into the mid scapular area. The minipumps released CDNF protein (rhCDNF, Batch 00400, 9.6 µg/µl, Biovian, Turku, Finland) (3 µg/24 h) (n = 26 for the male group, n = 14 for the female group) or phosphate-buffered saline (PBS) (n = 27 for the male group, n = 19 for the female group) for four weeks (Fig. 1). The cannula was fixed to the skull with Superglue Loctite 454 (Agnthos, Sweden). In the experiment, the mice were balanced into groups based on their rotarod motor performance before drug treatment. At 15 weeks of age of mice, the minipumps were removed, male mice were sacrificed after minipump removal, whereas females were sacrificed at 16 weeks of age (Fig. 1). This was based on the survival difference between genders (Suppl. Fig. 1).

### CDNF diffusion after chronic intrastriatal delivery

**Figure 2:**
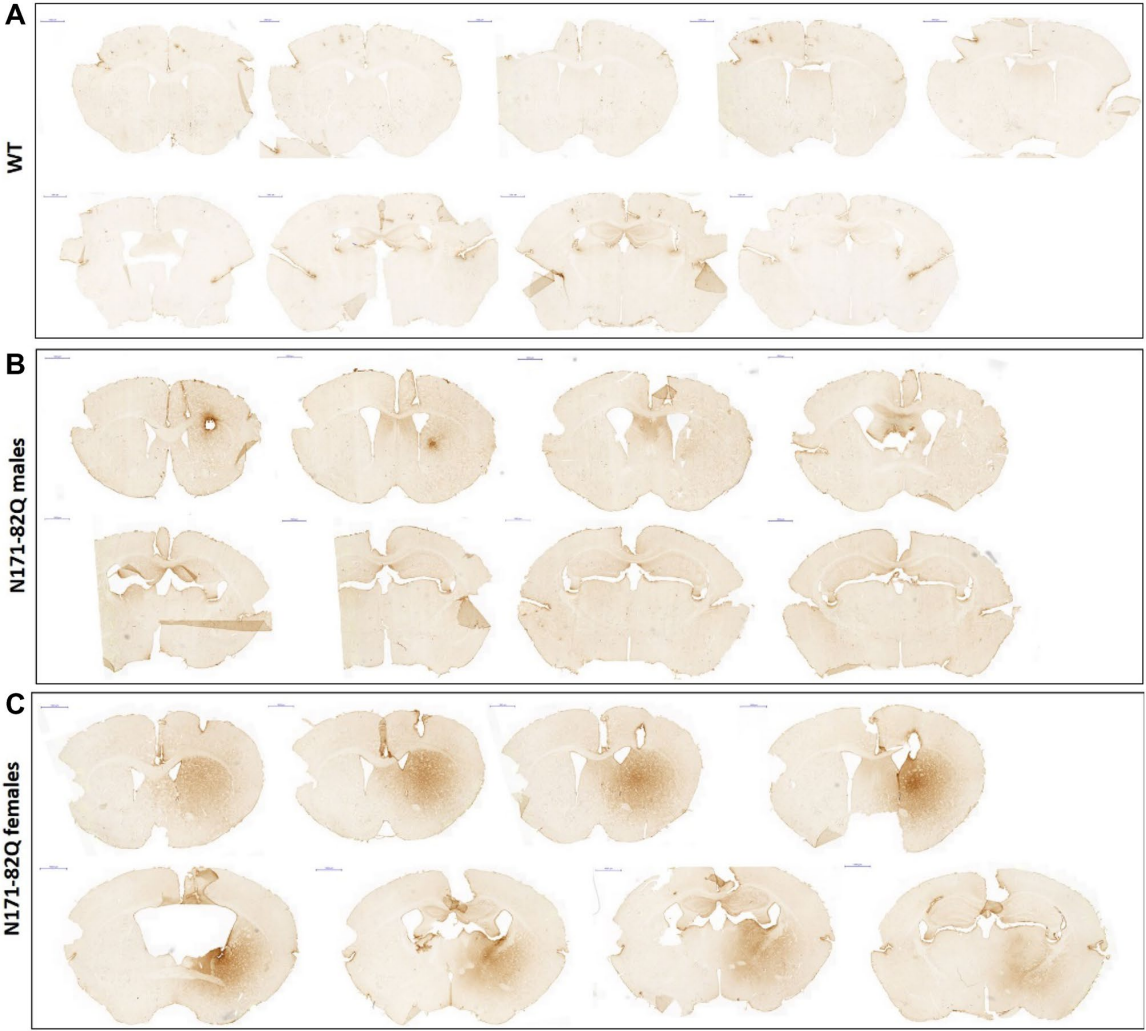
Diffusion of CDNF after chronic intrastriatal administration into N171-82Q mouse model (free-floating sections). Mice received an intrastriatal 4-week chronic infusion (A/P + 0.86; M/L − 1.8; D/V − 3.0) of CDNF (3 μg per 24 h). Immunohistochemical CDNF staining showed the CDNF distribution across the brain in WT animals (control, uninfused) (**A**), N171-82Q male mice (**B**), and N171-82Q female mice (**C**). Scale bar: 1000 μm.

In the diffusion experiment (Fig. 2), we used the tissue from mice N171-82Q and WT naïve mice, which received a chronic infusion of rhCDNF protein (3 µg/24 h) (human recombinant CDNF, Batch 00400, 9.6 µg/µl, Biovian, Turku, Finland) based on the protocol of chronic CDNF delivery, which was mentioned above. N171-82Q males (n = 2) were sacrificed right after the minipump stopped working (at 15 weeks age), whereas N171-82Q females (n = 4) were sacrificed one week later (at 16 weeks age). The analysis of diffusion was performed on tissue from a subset of animals from the CDNF infusion experiment. We used healthy naïve mice (n = 3) as a control for visual illustration of the CDNF diffusion.

### Behavioral experiments

Animal adaptation was performed when mice were 9 weeks of age; one week before baseline behavioral testing was done (10 weeks). Behavioral testing during the CDNF (or PBS) infusion period was performed weekly from 11 to 15 weeks of age for males or until 16 weeks of age for females. Untreated (no minipump installation) N171-82Q mice (n = 8 (males group) and n = 6 (females group) for Rotarod test; n = 4 (males group) and n = 5 (females group) for balance beam test) and wild-type mice (n = 13 (males group) and n = 6 (females group) for Rotarod test) were also used in behavioral tests as controls. The rotarod test system was used for measuring locomotion activity (Ugo Basile, Italy). The rotation speed was gradually increased from 4 to 40 rpm (revolutions per minute) over a maximum of 240 s. The average falling time was measured across trials, which were conducted twice per week. Moreover, balance motor coordination was analyzed by the balance beam walking test. Animals were placed on the round stick and were allowed to walk on it for a maximum of three minutes once per week. The travel distance and falling time were recorded. Any animals in poor condition or near the endpoint and who could not perform the behavioral tests were given zero scores. The digigait test (Ventral Plane Imaging (VPI) Technology, USA) was used as an additional motor test to check more specific stride/swing motor parameters. The speed of the treadmill (20 cm/s) was optimally chosen for all animals. Only animals who were able to perform the test over three-second intervals were used in the analysis. The hind limb clasping score was measured based on a previous study^40^.

### Immunostaining

After the behavioral experiments, mice were anaesthetized and perfused with PBS, followed by 4% paraformaldehyde (PFA) in PBS. Postfixed whole brains were stored in either 4% PFA or 4% PFA with further storage in 30% sucrose solution depending on the further procedure. The brains which were stored in 4% PFA were embedded in paraffin, further deparaffinized and cut into 10-µm-thick sections for immunohistochemical analysis; brains that were stored in sucrose solution were cut into 30 µm-thick free-floating sections and analyzed in the diffusion experiment.

#### CDNF immunohistochemistry

For these experiments, we used free-floating and paraffin-fixed sections for immunostaining and the ABC method. The free-floating sections (30 µm) were rinsed with PBS with following blocking in the mixture of 1% BSA, 0,2% Triton-×-100 in PBS for 1 h. Thereafter, sections were incubated in mouse anti-human CDNF antibody (mouse-hCDNF-6G5, stock solution 0.4 mg/ml, Icosagen, #302-100, 1:2000) overnight at 4 °C. The next day sections were incubated in anti-mouse biotinylated secondary antibody solution (1:400, Vector Laboratories, Burlingame, CA) for 1 h, followed by incubation in avidin-biotin-horseradish peroxidase complex (ABC) (Vestastain ABC kit, Vector) complex for 30 min. Staining was achieved with a 3,3′-diaminobenzidine tetrahydrochloride (DAB) solution (SK-4100, Vector DAB substrate kit).

Paraffin sections (10 µm) were deparaffinized by incubation of slides with sections in xylene for 4 × 5 min then 100% ethanol for 3 × 3 min, 95% ethanol for 3 min, 70% ethanol for 3 min, 50% ethanol for 3 min and deionized water (dH_2_O) for 3 min. Antigen retrieval was done by heating in 10 mM citrate buffer for 10 min and sections were rinsed using 1xPBS, and blocked with 1% BSA, 0.2% Triton-×-100 in PBS for 1 h and incubated overnight at 4 °C with mouse anti-CDNF antibody (mouse monoclonal, human CDNF, clone 6G5, Icosagen, 1:1000). Sections were washed and incubated with secondary antibodies (goat anti-mouse IgG, 1:400; Vector Laboratories, Burlingame, CA) for 1 h, and then for 30 min with ABC (Vestastain ABC kit, Vector) complex. Staining was achieved with DAB solution (SK-4100, Vector DAB substrate kit). After DAB staining slides with sections were incubated in 50% ethanol for 3 min, 70% ethanol for 3 min, 94% ethanol for 3 min, 100% ethanol for 2 × 2 min and in xylene 2 × 3 min for dehydration.

#### NeuN-, TH-, DARPP32- and parvalbumin immunohistochemistry

Immunostaining was performed on paraffinized brain sections using the ABC method. Sections were deparaffinized and incubated with 10 mM citrate buffer for 10 min as above, and nonspecific binding was blocked with 2% normal goat serum in TBS-T (1×TBS, 0.1% Tween-20). Following washing primary antibody was added overnight at 4 °C. These included NeuN antibody (mouse, 1:500 Chemicon, #MAB377), DARPP-32 (1:4000, rabbit, Abcam #ab40801), parvalbumin (mouse, 1:1000, Millipore #MAB1752), TH (mouse, 1:2000, Chemicon #MAB318). Sections were rinsed in TBS-T (1xTBS, 0.1% Tween-20) and incubated in biotinylated goat anti-mouse IgG (1:200 or 1:400 for DARPP-32; Vector Laboratories, Burlingame, CA) for 1 h at room temperature, followed by incubation for 30 min with avidin-biotin-horseradish peroxidase (ABC) complex. Staining was achieved with DAB solution (SK-4100, Vector DAB substrate kit). Further dehydration of slides was conducted. Slides with sections were placed into MilliQ followed by dipping in 50% ethanol for 3 min, 70% ethanol for 3 min, 94% ethanol for 3 min, 100% ethanol for 2 × 2 min and after in xylene 2 × 3 min). After dehydration coverslips with Depex mounting medium were placed on the glasses with sections. All sections were scanned with a Pannoramic 250 digital slide scanner (3DHISTECH, Hungary). Three to six striatal brain sections were used to measure immunoreactivity, which was quantitated relative to the control group. ImagePro was used to quantitate NeuN- and Parv-positive cells and all measurements were performed by personnel blinded to the treatment. ImageJ was used to analyze DARPP32- and TH- optical density, where corpus callosum area was used as background.

#### EM48 immunohistochemistry

We used EM48 antibody to detect mHtt aggregates, which targets the N-terminal region of human Htt (amino acids 1-212). The analysis was performed on the paraffinized brain Sections (10 µm) with the ABC method. After deparaffinisation, antigen retrieval and endogenous peroxidase inactivation, sections were incubated in TBS-T (1×TBS, 0.1% Tween20). Nonspecific binding was blocked by BSA (4% BSA, 0.3% triton-×-100, PBS). The sections were incubated with mouse EM48 (1:100, Millipore #MAB5374) for 48 h at 4 °C. After several washes in TBS-T (1×TBS, 0.1% Tween-20), the sections were incubated with goat anti-mouse secondary antibodies (1:200, Vector Laboratories, Burlingame, CA), followed by incubation for 30 min with ABC complex. Staining was developed with the DAB method with DAB substrate kit (SK-4100, Vector), then sections were dehydrated and mounted using cover slips. All sections were scanned with a Pannoramic 250 digital slide scanner (3DHISTECH, Hungary). Three to six striatal brain sections were used to measure immunoreactivity, which was quantitated relative to the control group (N171-82Q-PBS). Healthy animals (WT) did not have mHtt aggregates and were not used in the analysis. Aiforia Create version 5.0 (Aiforia Technologies Oy) was used to analyse EM48-immunoreactivity and inclusions.

### Deep learning neural network Aiforia analysis of mutant huntingtin aggregates

Neuronal cells and respective aggregates, in addition to tissue detection, were detected using artificial intelligence solutions by Aiforia Technologies Oy (Helsinki, Finland). This was achieved by implementing Aimodel/algorithm generated using Aiforia Create (Aiforia Technologies Oy; www.aiforia.com) version 5.0. Tissue regions were detected using semantic segmentation with complex mode and were a parent layer to the neuronal cell layer. EM48-immunoreactive mHtt aggregates were detected using object detectors (10 µm in diameter), and boundaries of the same were detected using the instance segmentation feature within Aiforia Create. EM48-antibodies labelled intranuclear inclusions were also detected using object detectors (5 µm in diameter) as a child layer to the cell body layer, which was set as the parent layer under which all aggregates were predicted. Boundaries of aggregates were also detected using instance segmentation. All the objects and instances for both neuronal cells (parent layer) and intranuclear aggregates (child layer) were set to very complex mode. Collectively the algorithm was generated with 2881 iterations, the algorithm was released, and data was analyzed in Aiforia Hub (Aiforia Technologies Oy). An example of the Aiforia detection system is shown in Fig. 6.

### Quantitative analysis of EM48 immunohistochemistry

Data was exported from Aiforia Hub, and further data processing was done by applying the following thresholds: the EM48-immunolabelled mHtt aggregates were in size range from 28 to 120 µm^2^, for intranuclear inclusions (between 1.5 and 8 µm^2^). Intranuclear inclusions were divided into groups based on area size and all inclusions smaller than 1.5 µm^2^ were counted as microaggregates and were not included in the final calculation of intranuclear inclusions. Quantitative data for analysis was performed as average across the area from several sections. The final analysis is performed as average between animals per each group and showed as density of EM48-positive cells and number of intranuclear inclusions separately in each region of interest.

### RT-qPCR

Reverse transcription quantitative polymerase chain reaction (RT-qPCR) was performed to analyze the ER stress markers and *BDNF* levels after chronic intrastriatal administration of CDNF in N171-82Q mice. Brain samples were placed into the mouse brain matrix for further sectioning, which helped to collect small brain regions by steel punch. Brain samples from the striatum and hippocampus were collected for further analysis. Total RNA was isolated from mouse brain tissue using TriReagent (Sigma Aldrich, MI, USA), and cDNA was made using Oligo(dT)18 primers with 1 µg, 500 ng or 200 ng RNA and Maxima H Minus Reverse Transcriptase (Thermo Fisher Scientific, MA, USA). cDNA was further diluted to 5 ng/µl, and 1 µl was used as a template in 10 ul total reaction for qPCR using the Lightcycler 480 SYBR Green I master mix (Roche, Switzerland) reaction kit. ER stress marker mRNAs (*GRP78, ATF4, ATF6, CHOP and XBP1S*) or *BDNF* mRNA and 5.8S reference gene expression were detected with Light Cycler® 480 Real-Time PCR System (Roche, Switzerland). The relative fold gene expression was calculated using the ∆∆Ct method. Results were expressed as a combination of female and male tissue, as there was no difference between genders. The list of primers is presented in Supplementary Fig. 2.

### Cell cultures and immunoblotting

Striatal cell lines derived from mice expressing different Htt genotypes and immortalized with a temperature sensitive large T antigen were used as previously described^41^. Control Htt cells expressed 7 polyglutamine (7Q) repeats while mHtt cells expressed 109 polyglutamine (109Q) repeats. The cells were cultured in Dulbecco’s Modified Eagle’s Medium (DMEM; Thermo Fisher Scientific) supplemented with 10% fetal bovine serum (FBS; Thermo Fisher Scientific), 100 mM L-glutamine (Thermo Fisher Scientific) and 100 mM penicillin–streptomycin (Thermo Fisher Scientific) at 33 °C in 5% CO_2_. For the experiments, the cells were incubated further for two days at 37 °C to induce neuronal differentiation. 100 ng/ml CDNF was then added for 24 h to half of the cultures, while the other half received vehicle only. Subsequently the cells were washed twice with ice-cold PBS and lysed in RIPA buffer (150 mM NaCl, 1% Triton-×-100, 0.5% sodium deoxycholate, 1% SDS, 50 mM Tris-HCl, pH 7.4) supplemented with protease inhibitors (COEDTAF-RO, Roche) and phosphatase inhibitors (PHOSS-RO, Roche). The lysates were centrifuged at 16,200×*g* for 15 min at 4 °C, the supernatants were collected, and the protein concentrations were measured using Pierce BCA Protein Assay Kit (Thermo Fisher Scientific).

Equal amounts of protein (40 µg) were separated by SDS-PAGE and transferred onto nitrocellulose membranes as previously described^42,43^. The membranes were washed with TBS-T (50 mM Tris-HCl pH 7.5, 150 mM NaCl, 0.1% Tween 20), blocked for 1 h in 5% skimmed milk diluted in TBS-T, and incubated with primary antibodies against BDNF (1:500, Icosagen, #329-100) or GAPDH (1:10,000, Millipore, #MAB374) overnight at 4 °C with gentle agitation. Following washes with TBS-T, the membranes were incubated with horseradish peroxidase-conjugated secondary antibodies (1:2500, Jackson ImmunoResearch Laboratories, UK) for 1 h at room temperature with gentle agitation. Proteins were detected by enhanced chemiluminescence using Pierce ECL Plus Western Blotting Substrate (Thermo Fisher Scientific) and imaged on FLA-9000 Starion scanner (Fujifilm Life Sciences, MA, USA). The immunoblots were quantified with ImageJ quantification software (NIH, MD, USA).

### Statistical analyses

The results were analyzed with repeated‐measures ANOVA and mixed-effects ANOVA or unpaired/paired t‐test for behavioral tests. One-way ANOVA was used for the analysis of three and more groups. The Wilcoxon matched-pair signed-rank and the Kruskal–Wallis tests were used to analyze the percentage of animals with clasped hindlimbs. The survival test was analyzed with the log-rank Mantel-Cox test. Immunological results were analyzed by ANOVA and Student’s t-test, and RT-qPCR data by unpaired t-tests. Results are shown as means ± SEMs and considered significant at a value of *p* ≤ 0.05. All statistical tests and graphs were performed in GraphPad Prism software version 7.02. (GraphPad, CA, USA, www.graphpad.com).

## Results

### Diffusion of CDNF in brain tissue of N171-82Q mice

The diffusion of CDNF in brain tissue after chronic intrastriatal infusion was characterized in N171-82Q mice (Fig. 2), and in WT mice not receiving CDNF were used as controls (Fig. 2A). Immunohistochemical CDNF staining illustrates the distribution of CDNF across the brain in the N171-82Q mouse model. As shown in Fig. 2B,C the diffusion of CDNF had a gender diversity with female having a more extensive diffusion pattern. N171-82Q male mice showed very small diffusion area, which was visible only near the injection site. CDNF diffusion was visible across the brain in N171-82Q females, from the site of injection rostrocaudally. In these experiments, the endpoints of analyses in the two genders were different based on the progression of symptoms. Thus, the N171-82Q males were sacrificed one week earlier than females at 15 weeks of age, whereas the females were sacrificed at 16 weeks of age, which may account for this difference observed (Fig. 1).

### CDNF postpones the onset of symptoms and improves motor coordination in the N171-82Q transgenic mouse model

**Figure 3:**
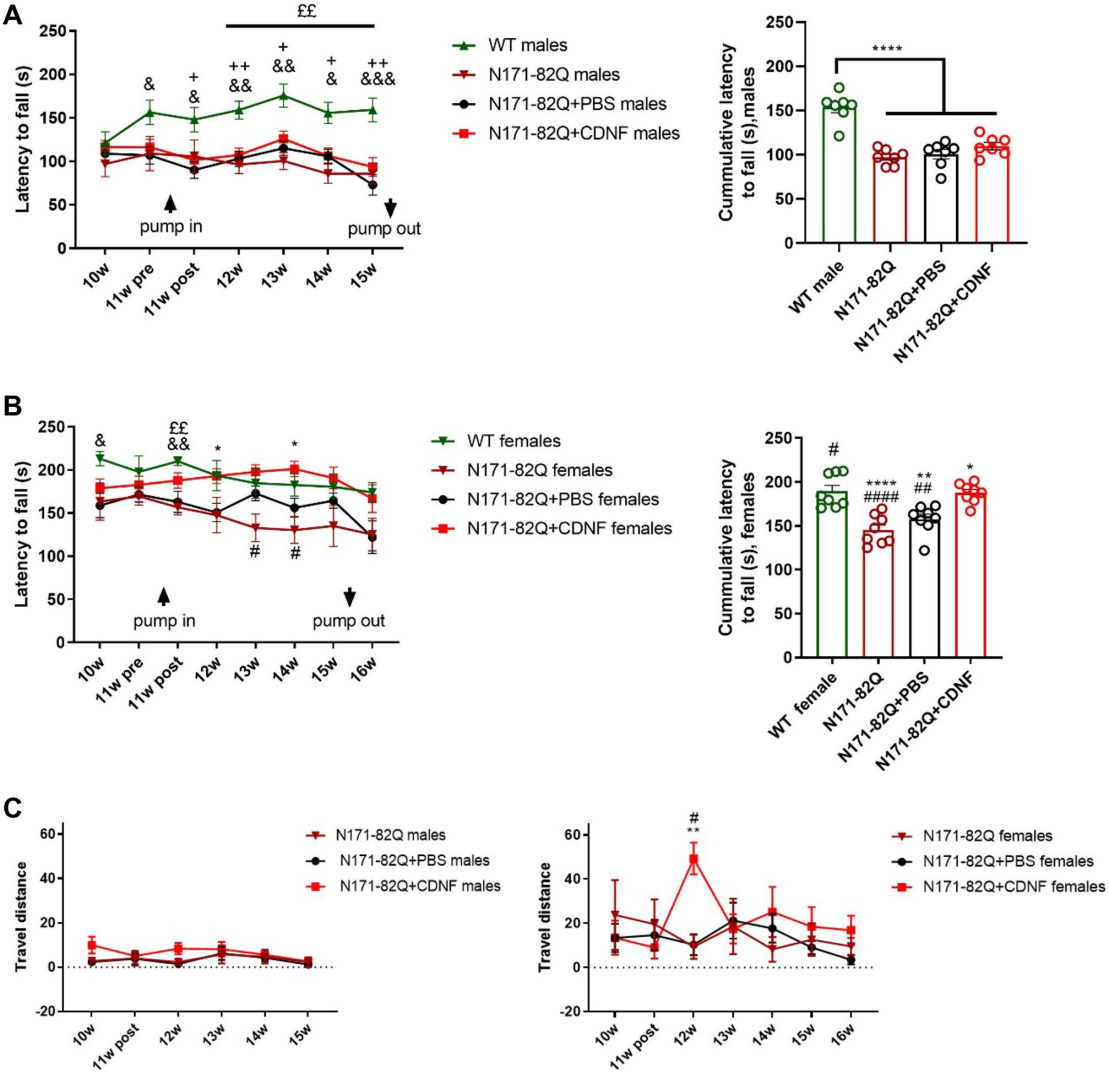
Motor presentation. Mice received a chronic infusion of CDNF- or PBS- into the right striatum. (**A**) Rotarod in N171-82Q male mice. There was not a significant improvement in motor performance between PBS-treated, CDNF-treated and untreated N171-82Q males. Healthy animals showed a significant difference from N171-82Q animals in motor performance and cumulative latency. &, comparison between WT and N171-82Q + PBS males; + , comparison between WT and N171-82Q + CDNF males; £, comparison between WT and untreated N171-82Q males. Values are expressed as group mean ± SEM; *p < 0.05, **p < 0.005, ***p < 0.0005, ****p < 0.0001; one-way ANOVA test. N number = 8–26 (N171-82Q groups) and n = 13 (WT group). (**B**) Rotarod in N171-82Q female mice. CDNF-treated N171-82Q females showed a statistically significant effect on motor coordination compared to vehicle-treated groups and untreated N171-82Q animals. Cumulative results showed a significant improvement in motor coordination in CDNF-treated and healthy animals compared to PBS-treated and untreated N171-82Q females. *, comparison between N171-82Q + CDNF and N171-82Q + PBS females; #, comparison between N171-82Q + CDNF and untreated N171-82Q females; &, comparison between WT and N171-82Q + PBS females; £, comparison between WT and untreated N171-82Q females. Values are expressed as group mean ± SEM. ****p < 0.0001, **p < 0.01, *p < 0.05, repeated-measures ANOVA test, one-way ANOVA test. N number = 6–19 (N171-82Q groups) and n = 6 (WT group). (**C**) Travel distance in the balance beam test. The CDNF-treated female group showed a statistically significant positive effect on balance performance one week after minipump installation in comparison with the vehicle group. Values are the means ± SEMs. **p < 0.01, *p < 0.05, mixed-effect ANOVA test, Sidak’s post hoc test. N number per group = 4–19.

We next examined the effects of chronic CDNF infusion on behavior of N171-82Q mice. There was a significant weight loss between WT and untreated N171-82Q mice, CDNF- and PBS-treated male and female groups; however, no difference between CDNF- and PBS-treatment groups was found (Suppl. Fig. 3). We conducted the rotarod test twice per week to assess motor deficits, and the average between two-time points was used in the analysis. The results of the rotarod performance demonstrated a significant effect only in motor presentation and cumulative latency to fall between WT animals and N171-82Q male groups, but there was no effect between treatment groups (one-way ANOVA, Tukey’s post hoc test *p* < 0.0001) (Fig. 3A). A significant drug treatment effect was observed between N171-82Q females in the PBS- and CDNF-treated groups in motor performance (two-way repeated-measures ANOVA, treatment × time interaction effect f_21,287_ = 1.091, *p* = 0.3572; time effect f_4.680,191.9_ = 3.411, *p* = 0.0068; treatment effect f_3,41_ = 4.390, *p* = 0.0091) (Fig. 3B). CDNF-treated female mice showed significantly higher cumulative latency to fall compared to untreated and PBS-treated transgenic females (one-way ANOVA, Tukey’s post hoc test *p* < 0.0001, *p* < 0.005) (Fig. 3B). Moreover, we examined balance performance as an additional motor assessment test. The balance beam test showed no difference in travel distance and falling time between the transgenic male groups (Fig. 3C). Furthermore, a significant improvement in balance, analyzed by travel distance in the balance beam test, was noted in the CDNF-treated female group during the first week after minipump installation (mixed-effect ANOVA test, Sidak’s post hoc test *p* < 0.01, *p* < 0.005) (Fig. 3C).

**Figure 4:**
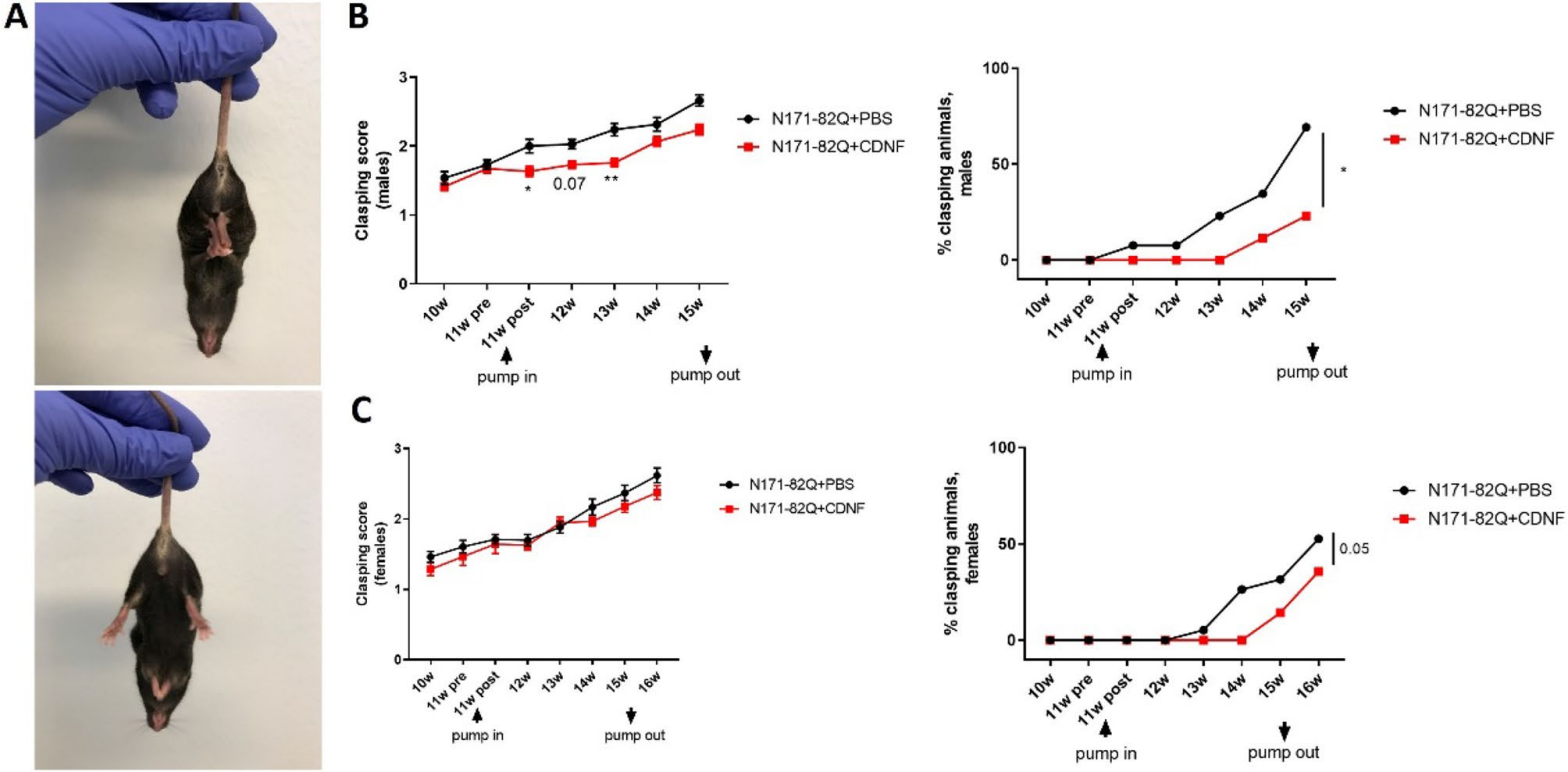
Clasping hindlimbs of N171-82Q mice. (**A**) Clasped hindlimbs (top photo) and unclasped hindlimbs (bottom photo). (**B**) Clasped hindlimbs in male mice. The CDNF-treated group presented statistically lower clasping scores than the vehicle group. *p < 0.05, **p < 0.005, Kruskal–Wallis test, Dunn post hoc. The percentage of animals with totally clasped hindlimbs in the CDNF-treated group was significantly lower than in the control group. *p < 0.05, paired t-test. (**C**) The clasping score in female groups did not show a difference. The number of females with totally clasped hindlimbs was significantly lower compared to the control. *p* = 0.05, paired t-test. Values are the means ± SEMs. N per group = 14–26.

The clasping of hindlimbs was tested as indicated (Fig. 4A). Clasping usually increases during the development of pathology in HD mouse models^40^. The clasping score was significantly decreased (Kruskal–Wallis’s test, Dunn’s post hoc test *p* < 0.005, *p* < 0.05) in the CDNF-treated males compared with PBS group (Fig. 4B), while females did not show significant differences in clasping score progression (Fig. 4C). We further observed the number of animals with totally clasped hindlimbs, which revealed that the percentage of males with totally clasped hindlimbs in the CDNF-treated male group was significantly decreased compared to the vehicle group (paired t-test, *p* < 0.05) (Fig. 4B). Additionally, CDNF-treated females showed a positive tendency to a decrease in the number of clasping animals (paired t-test, *p* = 0.05) (Fig. 4C).

Taken together, the results with the behavioral tests reveal that CDNF has a positive effect on motor coordination in the N171-82Q mice compared with corresponding PBS treated mice.

### The effect of CDNF on nerve cell immunoreactivity and brain weight in N171-82Q mice

**Figure 5:**
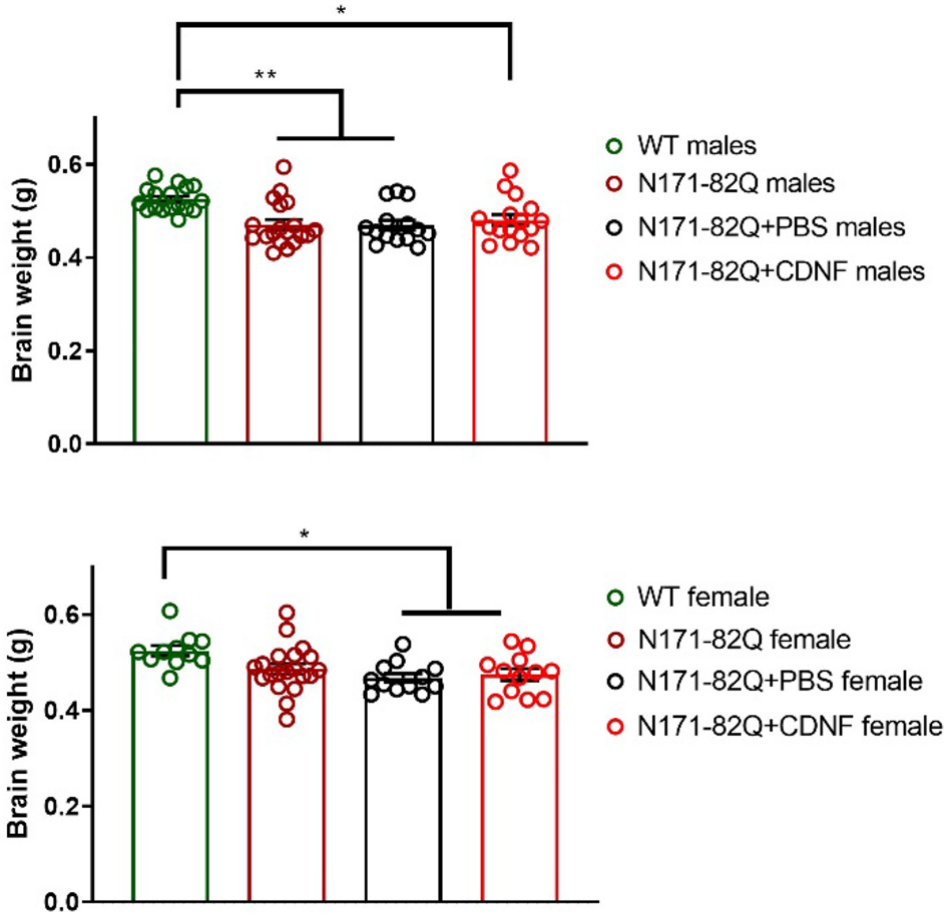
Effect of chronic CDNF administration on brain weight of N171-82Q mice. CDNF did not affect brain weight compared to vehicle in two genders. Values are means ± SEM. *p < 0.05, **p < 0.001, one-way ANOVA, Turkey’s post hoc test. N number per group = 11–20.

To study the effects of CDNF in more detail we performed immunohistochemistry using brain sections from mice. We observed no differences in NeuN-, Parv- and DARPP-32- immunoreactivity after CDNF administration in the N171-82Q mice at 11 weeks (Suppl. Fig. 4). Moreover, the TH optical density in the striatal area of N171-82Q mice was not altered by CDNF (Suppl. Fig. 4). The endpoint for this experiment was 15 weeks for males and 16 weeks for females that may have been too early to observe any significant changes in immunostainings. On the other hand, analyzing the size of the brain demonstrated a smaller brain weight of N171-82Q mice compared with WT (Fig. 5). The average difference in the brain weight between WT and N171-82Q CDNF-treated male animals was slightly smaller than the difference between WT animals and N171-82Q PBS-treated male group (Fig. 5). On the other hand, N171-82Q females had a similar reduction in brain weight in both CDNF- and PBS-treatment groups compared with WT (Fig. 5). We further noticed that the volume of the striatum, cortex and piriform cortex showed no significant difference between PBS- and CDNF-treated groups (Suppl. Fig. 5).

### Diversity of EM48-immunoreactive mHtt aggregates in control and N171-82Q mice

**Figure 6:**
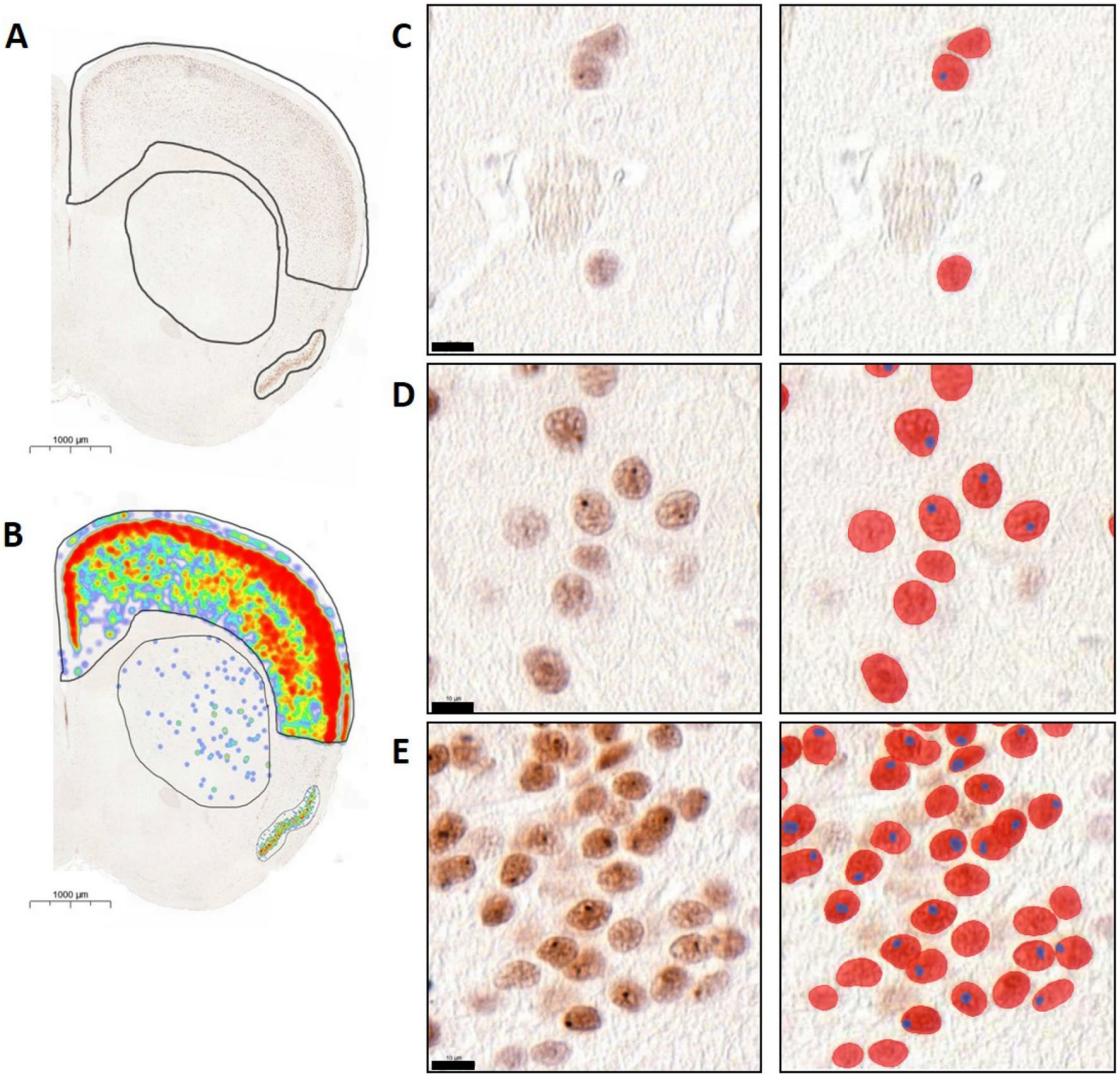
Analysis for EM48-immunopositive mutant huntingtin aggregates using Deep neural network based Aiforia platform. Representative image of a single N171-82Q animal (male, 23 weeks age old). (**A**) Areas (neocortex, striatum and piriform cortex) of the N171-82Q mouse brain, which were analyzed using Aiforia. (**B**) Heatmap of analysis outputs, showing the expression profile distribution of EM48-immunopositive mutant huntingtin aggregates in the respective analysis regions (**A–B**) Magnification: 1.0×. Scale bar = 1000 μm. (**C–E**) High power zoom of the mHtt aggregates. Red-colored is the area of EM48 positive mHtt aggregates, blue-colored is the area of EM48-labeled intranuclear inclusions. (**C**) striatal area, (**D**) neocortex area, (**E**) piriform cortex area. (**C–E**) Magnification 100.0×. Scale bar = 10 μm.

To evaluate the effect of CDNF treatment on mHtt aggregation, we first analyzed the distribution of the latter using a deep neural network-based Aiforia platform (see Fig. 6A,B) in untreated N171-82Q mice. Algorithm was generated with 2881 iterations and released when false-negative and false-positive error rates were 0.05 and below.

For this, EM48-positive cells were analyzed in three brain areas: striatum (Fig. 6C), cortex (Fig. 6D) and the piriform cortex (Fig. 6E). We observed two types of aggregates: neuropil and nuclear inclusions, but the neuropil aggregates were challenging to calculate. Using the AI-algorithm we could detect EM48-positive cells (red color cells in Fig. 6C–E) and intranuclear inclusions (blue color in Fig. 6C–E). No EM48-positive aggregates were detected in wild-type control, and these animals were omitted from the analysis (data not shown). For our analysis we used untreated N171-82Q mice at different ages: 10 (n = 4), 12 (n = 5), 14 (n = 3), 16 (n = 4), 19 weeks (n = 4) and more than 19 weeks (20–30 weeks, n = 5).

**Figure 7:**
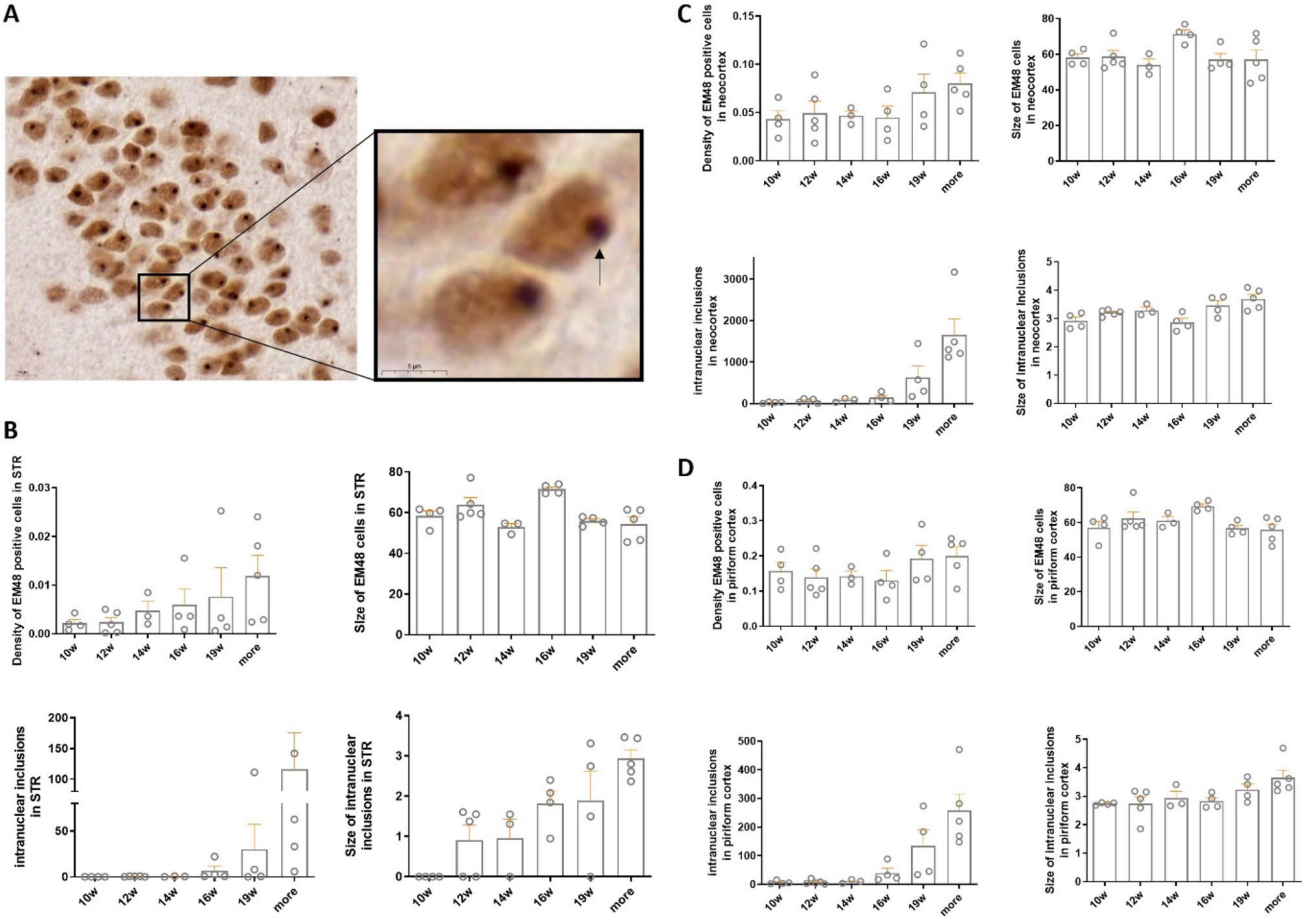
Characterization of development of mHtt aggregates in the N171-82Q mouse model. (**A**) EM48-positive aggregates in N171-82Q mouse model. The black arrow points to the intranuclear inclusion. Magnification: 100×, scale bar = 10 μm. Magnification: 300×, scale bar = 5 μm. Appearance of EM48 positive aggregates in N171-82Q mice was analyzed in six points:10, 12, 14, 16, 19 and more than 19 weeks (between 20 to 30 weeks). (**B**) Development of huntingtin aggregates in striatum. The number of EM48 positive cells, intranuclear aggregates, and size of intranuclear inclusions increased in an age-dependent manner. (**C**) Formation of EM48-labelled aggregates in the neocortex. The number of intranuclear aggregates also increased in the neocortex in an age-dependent manner. (**D**) The number of intranuclear aggregates enhanced in the piriform cortex in an age-dependent manner. The values are expressed as means ± SEM. N = 3–5 animals per group.

We observed that N171-82Q mice had an age-dependent appearance of EM48-positive staining in the striatum and neocortex (Fig. 7B,C), with an increased number of intranuclear EM48-positive mHtt aggregates (see Fig. 7A) that are present in the striatum, neocortex and piriform cortex (Fig. 7B–D). Moreover, a similar tendency was found with regard to the size of intranuclear aggregates in the striatum (Fig. 7B) and a slight increase in the piriform cortex area (Fig. 7D). The most apparent changes of aggregates, however, were observed in the striatum (Fig. 7B). These observations were not statistically analyzed because of the small number of animals used.

### Effect of CDNF on mutant huntingtin aggregates in N171-82Q mice

**Figure 8:**
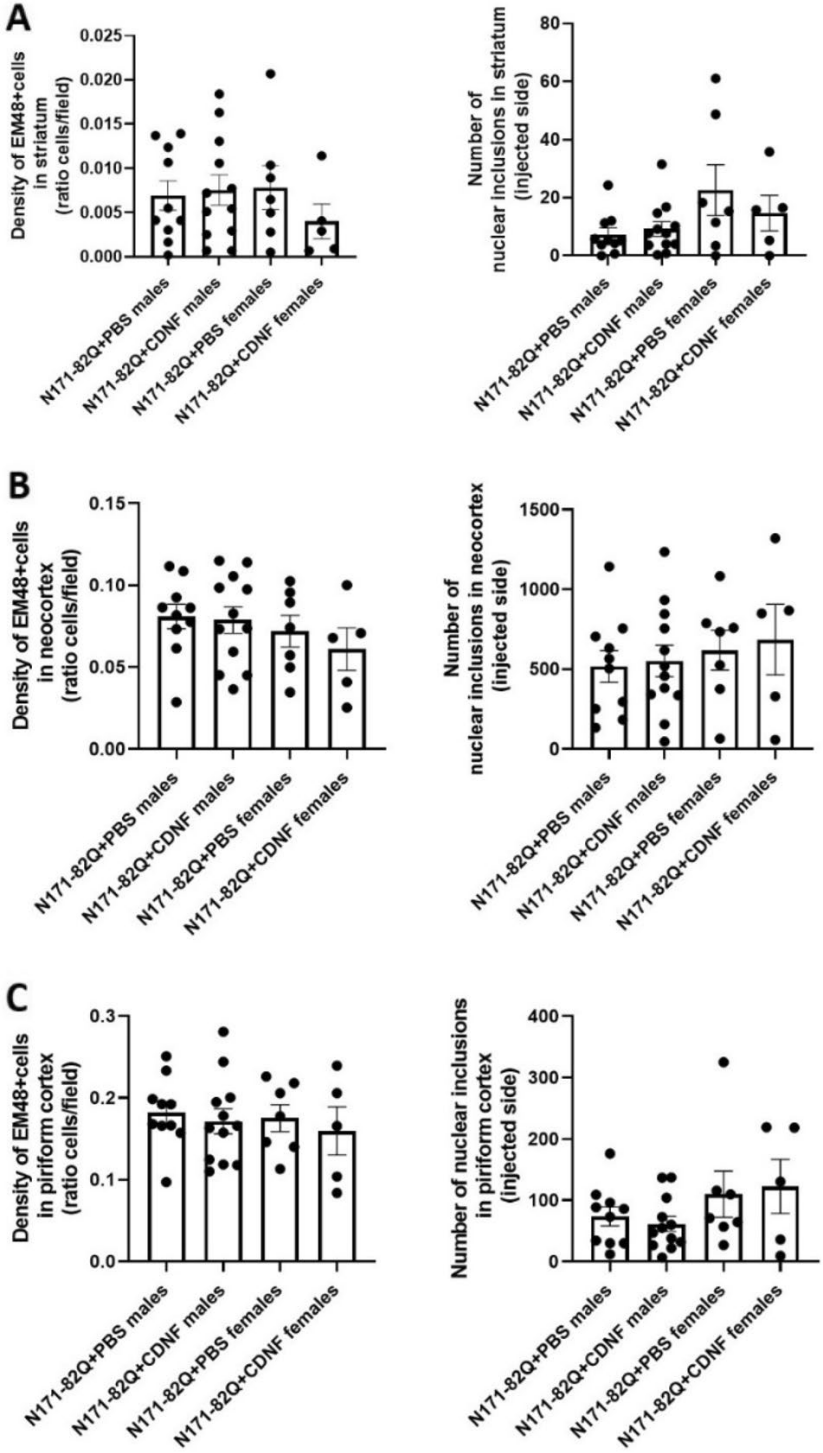
EM48-positive staining and intranuclear inclusions in the N171-82Q mouse model after chronic injection of CDNF or PBS. WT animals did not show the presentation of mHtt inclusions. The density of EM-positive nuclear staining and number of inclusions in the striatum (**A**), neocortex (**B**) and piriform cortex (**C**) of N171-82Q mice. Quantification analysis was performed at week 15 of life for males and 16 for females. Values are means ± SEM. N number per group = 5–7 (females) and 10–12 (males).

To analyze the effects of CDNF on EM48 staining and intranuclear aggregates in N171-82Q mice we studied mainly the striatum, neocortex and piriform cortex, albeit there were also aggregates present in the hippocampus (data not shown). Whereas CDNF-treatment showed a tendency to reduce the number of EM48-positive staining in the striatal area of N171-82Q females (Fig. 8A) this was not the case for N171-82Q males. Moreover, CDNF did not significantly reduce the number of mHtt inclusions in the striatum of N171-82Q females, neither was the size of mHtt inclusions different between groups (Fig. 8A). This was also the case when analyzing aggregates in the neocortex of female and male N171-82Q mice (Fig. 8B). There was, however, a slight decrease in mHtt intranuclear inclusions after CDNF treatment in the piriform cortex of N171-82Q males (Fig. 8C), but this difference was not statistically significant. In addition, we observed a gender difference in the N171-82Q mice with respect to the amount of intranuclear inclusions in the striatum and the piriform cortex, with males having almost twice less inclusions compared with females (Fig. 8A,C). From the data obtained using the deep-learning approach it was further seen that CDNF did not affect the size of EM48-positive cells.

Collectively, our results revealed that chronic striatal CDNF administration did not significantly reduce the amount of mHtt inclusions in the striatum of N171-82Q female mice under the present conditions. On the other hand, in the piriform cortex of CDNF-treated N171-82Q male mice, there was a decrease in the number of intranuclear mHtt inclusions compared with controls. However, this difference was not statistically significant. The obvious lack of an overall effect of CDNF on mHtt aggregates in other brain regions could be due to the relative concentration of CDNF present at the different locations and times in brain after infusion. This is an important issue warranting further investigations.

### CDNF increases BDNF mRNA levels in vivo in N171-82Q mice

**Figure 9:**
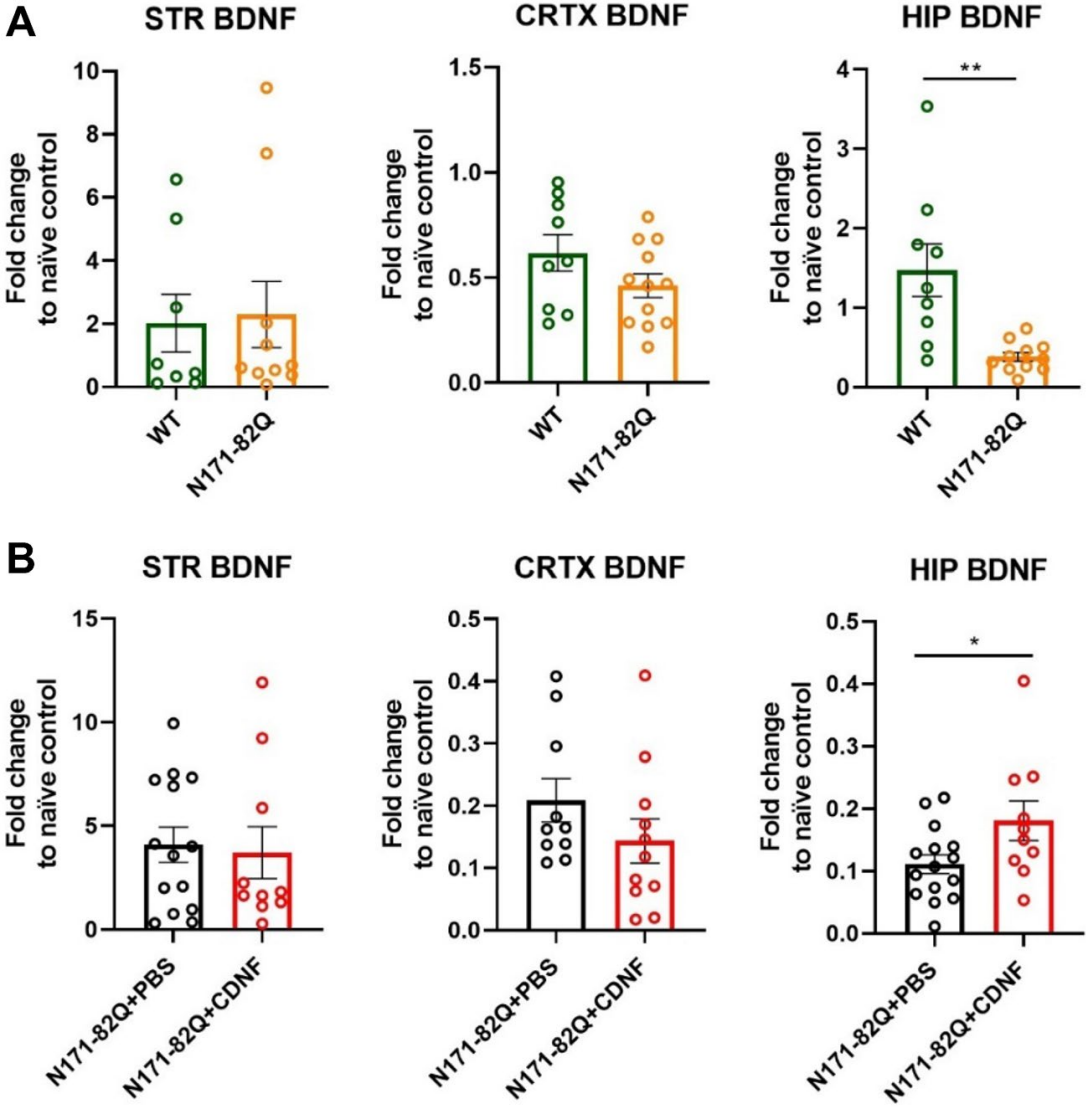
mRNA level of *BDNF* in the striatum, cortex and hippocampus. (**A**) mRNA expression of *BDNF* in total lysates of striatum, cortex and hippocampus; results are presented as a fold change compared to naïve control. **p < 0.005, unpaired t-test. Values are the means ± SEMs. N per group = 8–11. (**B**) qPCR for *BDNF* mRNA in the striatal, cortical and hippocampal extracts of N171-82Q mice chronically treated with CDNF or PBS. Results are presented as a fold change compared to naïve control. *p < 0.05, unpaired t-test. Values are the means ± SEMs. N per group = 10–15.

While studying gene expression levels in the N171-82Q mice, we observed that the *BDNF* mRNA levels were significantly decreased (unpaired t-test, *p* < 0.005) in the hippocampus of these mice compared with WT animals (Fig. 9A). We therefore examined whether the chronic CDNF infusion may also affect the *BDNF* mRNA levels. *BDNF* mRNA levels were significantly higher in the hippocampus of N171-82Q CDNF-treated mice (unpaired t-test, *p* < 0.05) when compared with the PBS-treated group, suggesting that CDNF may partially rescue the loss of BDNF in N171-82Q mice (Fig. 9B). Interestingly, in these experiments we noted no decrease in *BDNF* mRNA level in the striatum and cortex of untreated N171-82Q mice (Fig. 9).

### Effects of CDNF on BDNF in cultured striatal neurons

**Figure 10:**
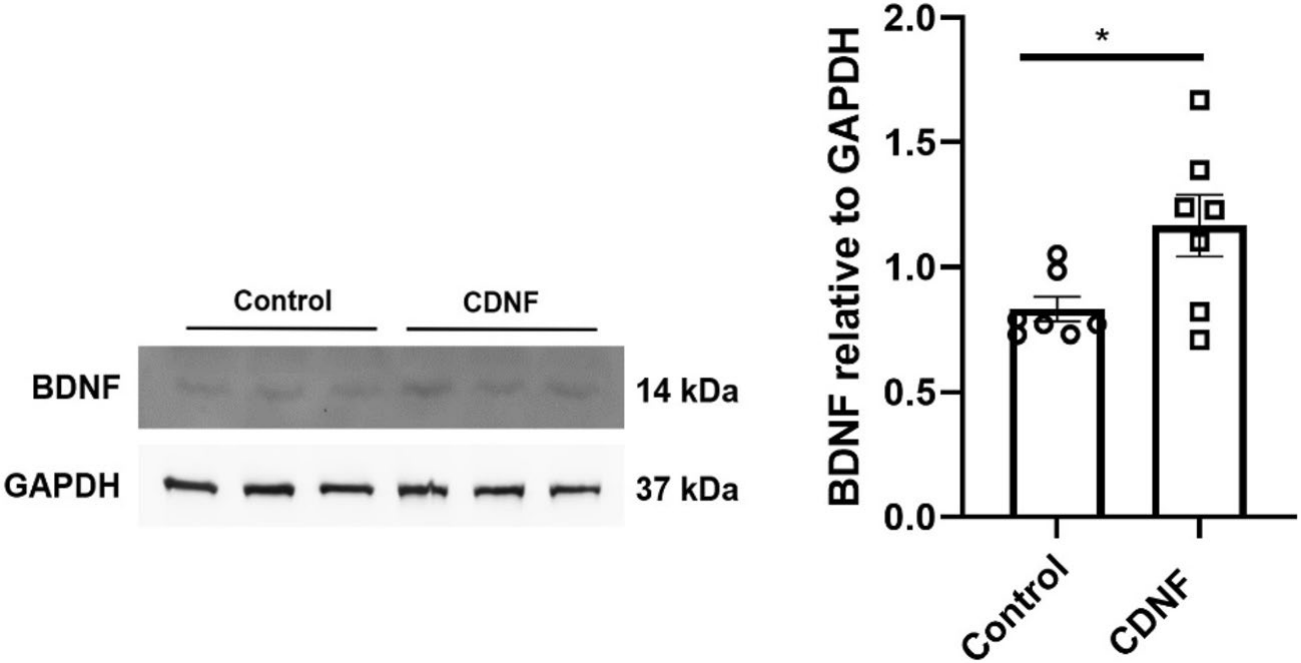
CDNF increases the levels of mature BDNF in striatal neurons expressing mutant huntingtin protein. Striatal neurons expressing mutant huntingtin were treated for 24 h with 100 ng/ml CDNF or vehicle. Cell lysates were subjected to immunoblotting for BDNF, using GAPDH as loading control. Left, immunoblot. Right, histogram with quantification of the relative ratio of BDNF compared with GAPDH. Values represent mean ± SEM, *p < 0.05, unpaired t-test, n = 7.

To study whether CDNF indeed can affect BDNF levels in neurons, we studied cultured striatal neurons expressing mutant Htt with 109Q repeats. Cells incubated in vitro were stimulated for 24 h with 100 ng/ml CDNF followed by immunoblotting as described in Methods. Results showed that CDNF increased the level of mature BDNF (14 kDa) in these cells compared with cells treated with vehicle only (unpaired t-test, *p* < 0.05; Fig. 10). Interestingly, there was no effect of CDNF on BDNF levels in striatal neurons expressing 7Q repeats containing Htt (data not shown). Together these results show that CDNF can increase BDNF in mHtt expressing striatal neurons in culture, which may contribute to its beneficial effects in the neurons^23^.

### CDNF effects on ER stress markers in the striatum of N171-82Q mice

**Figure 11:**
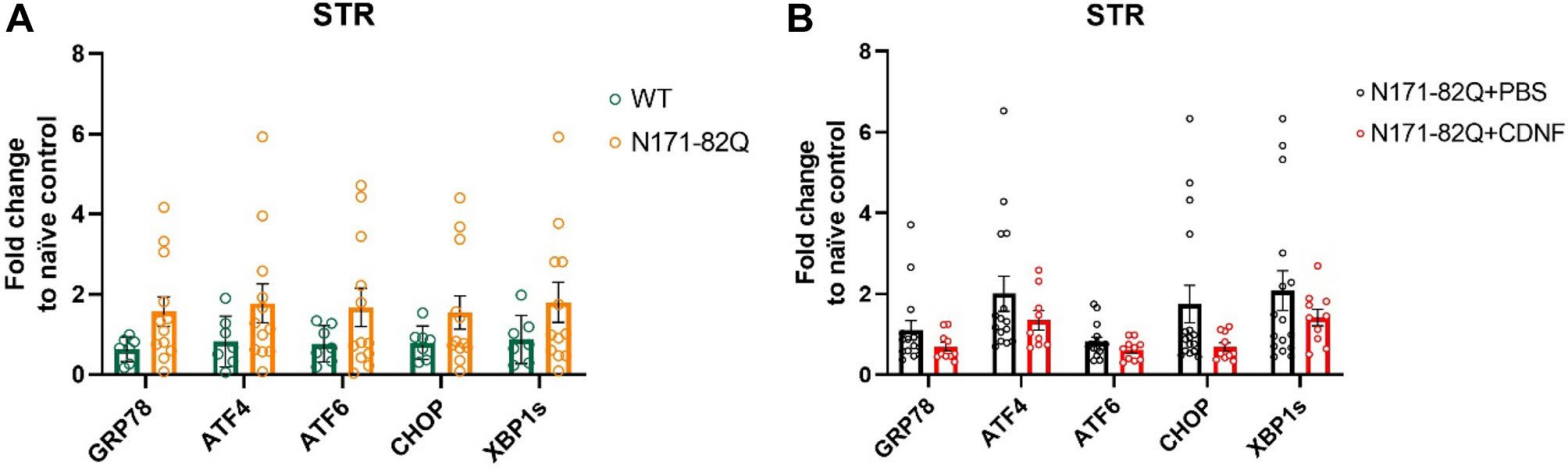
RT-qPCR analysis of ER stress markers in the N171-82Q mouse model. (**A**) ER stress marker levels in the striatum (STR). (**B**) ER stress marker levels in the striatum of CDNF- and PBS- treated mice. Values are the means ± SEMs. Unpaired t-test. N per group = 10–15.

Next we analyzed whether CDNF influences the mRNA levels of *GRP78* (alias *BIP*), *ATF4, ATF6, CHOP and XBP1S* in the striatum of healthy controls and N171-82Q mice. Data showed that there was an increase in the expression of ER stress markers in the N171-82Q mice regardless of their gender compared with WT animals at endpoint based on their wellbeing, however, the changes in ER stress markers were not significantly different in N171-82Q mice as compared with WT mice (Fig. 11A). CDNF treatment reduced the level of all five ER stress markers on average compared to PBS, however this difference was not statistically significant (Fig. 11B). However, there was a tendency for reduced *CHOP* expression in the N171-82Q striatum after CDNF infusion (unpaired t-test, *p* = 0.08) (Fig. 11B). A similar trend was also seen for *GRP78, ATF4, ATF6 and XBP1s* expression after CDNF-treatment, although the data did not reach statistical significance (Fig. 11B). These findings may be related to the relatively low number of animals used in these experiments.

## Discussion

As a background for the present study, we recently reported a positive effect of intrastriatal CDNF injection in the QA toxin rat model of HD. We found that CDNF is neuroprotective against QA toxicity in vitro and in vivo*,* and CDNF may also promote neurogenesis in vivo^23^. Previously, CDNF has been shown neuroprotective effects in different neuropathological conditions, such as in PD^27,37,38,44^, Alzheimer’s disease (AD) and amyotrophic lateral sclerosis (ALS)^24^.

Regarding the amount of protein used in in vivo experiments, several aspects were considered. In the 6-OHDA rat model of PD, chronic CDNF infusion (3 µg/day; 3 continuous days) improved motor imbalance and protected DA neurons^37^.﻿ In the TDP-43 trangenic rat model of ALS, chronic 4-week CDNF intracerebroventricular infusion (6 µg/24 h) had a positive effect on motor coordination and protected motoneurons in spinal cord^24^. Importantly, Garea-Rodriguez et al.^39^ revealed a significant increase of dopamine transporter binding activity in lesioned animals treated with CDNF as compared to vehicle-treated lesioned animals^39^. The outcome from clinical trials of PD patients conducted by Herantis Pharma showed pronounced DAT PET response in PD patients receiving a mid-dose CDNF compared to the high-dose group^47^.

Based on the above findings and previous application of the same dose (3 µg/day; 1-month infusion) in the mouse model of ALS (De Lorenzo, doctoral thesis ISSN 2342-317X), we chose here to use a dose of 3 µg per day of recombinant human CDNF (rhCDNF) for our experiments. For this, we employed the N171-82Q transgenic mouse HD model that shows development of motor deficits, brain atrophy, and accumulation of mHtt aggregates, which comprise the significant signs of HD pathology^5^. Important to note that the N171-82Q mouse model has wide phenotypical variability, thus it is necessary to include enough animals for the preclinical studies^9^. Previous studies have also shown that the phenotypes of N171-82Q females and males can differ^48–50^. In support of this, our present findings revealed a gender difference in motor coordination in the N171-82Q mice. Likewise, we found that the survival of N171-82Q male mice was shorter compared with N171-82Q females. This was also taken into account in the choice of endpoints used for studies of male and female N171-82Q mice.

We detected a significant difference between the effects of CDNF on the motor coordination and balance of N171-82Q mice compared with controls. Interestingly, CDNF treatment increased muscle strength in the rotarod test in N171-82Q females but not in males. Evaluation of balance coordination further showed that CDNF-treated N171-82Q females displayed longer travel distances than the vehicle group, but no difference was found for male mice. Likewise, CDNF did not affect the weight loss and stride length of both N171-82Q mice genders (Suppl. Fig. 3).

Most importantly, we observed a beneficial effect of CDNF on the clasping of hindlimbs in both genders, with N171-82Q males revealing a significantly lower clasping score after CDNF treatment. Notably, N171-82Q females showed a decrease in the number of animals with clasped hindlimbs even after the minipump’s removal, which indicates the prolongation of the positive effect of CDNF in the brain tissue. These results could be explained by the wider and longer diffusion of CDNF in the brain tissue of N171-82Q female mice (Fig. 2). The reason of the large difference in CDNF diffusion between both sexes can be explained by difference in the degradation of CDNF, however fundamental basis of it is unclear and warrants further investigations.

Furthermore, we observed that brain weight was higher on average in the CDNF-treated N171-82Q males in comparison with PBS-treated or untreated N171-82Q males (Fig. 5). However, immunostaining of the brain sections revealed no difference in NeuN- or in Parv- and DARPP-32 immunoreactivity between the groups. It is possible that the endpoint chosen for these experiments was too early to detect any significant neuronal loss, therefore additional studies using later endpoints are warranted.

The accumulation of mHtt aggregates is usually considered as a hallmark for the progression of HD. The prevention of mHtt aggregation can also be a potential therapeutic target for slowing symptoms or inducing recovery during the disease. However, the precise physiological role of mHtt aggregates is still under debate and could change during the course of the disease^51^. As shown for other neurodegenerative diseases, different types and stages of protein aggregates can be present in vulnerable cells underlying the disease process. Previously, it was suggested that only the monomeric or oligomeric stage of mHtt and not large visible aggregates could cause ER stress linked to the disease^52^. In line with this, it was reported that the oligomeric, non-aggregated form of mHtt is the most toxic species^53^, and that the formation of mHtt aggregates might be a coping mechanism by neurons to reduce mHtt toxicity.

Previous studies have shown that the expression of mutant N-terminal fragments of mHtt protein in neuronal cells induces the formation of aggregates with increased cell toxicity^54^. It was shown that the localization of EM48 aggregates show gradual increase of nuclear aggregates formation from early to the late stages of HD progression in the cortex in postmortem tissue and opposite tendency in the striatum area^55^. The formation of EM48-positive aggregates was found in the striatum and cortex of N171-82Q mice with no presentation in white matter^12^, while the detailed detection and progression of EM48 aggregates in these areas in N171-82Q have not been highlighted. In the present study, we examined the development of EM48-positive aggregates in vivo in the N171-82Q mouse model of HD using a novel approach based on a deep learning method. We observed a gradual age-dependent increase in the number and size of EM48-positive cells and intranuclear inclusions in the striatum, neocortex and piriform cortex in N171-82Q mice. These findings indicate a correlation between the deterioration of motor behavior observed in these mice and the appearance of the mHtt aggregates. In support of this, there is an increase in EM48-positive inclusions found in old compared with young mice in another mouse model of HD, the R6/1 mice at the time of motor deficits appearing^56^.

Employing an unbiased AI-based method for aggregation detection, we then investigated whether CDNF treatment in vivo would influence mHtt aggregation. We hypothesised that CDNF could possibly be involved in the degradation or the prevention of mHtt aggregates formed by activating the proteasome or autophagy in neurons. Data obtained showed that there was a trend towards a decrease in the density of EM48-immunoreactivity and the number of intranuclear inclusions in the striatum of N171-82Q female mice. In contrast, this was not apparent in other brain areas examined. The failure to reach statistical significance in these studies could be due to the low number of mice analysed or the inaccuracy in detecting the real toxic species of mHtt that may be affected by CDNF. Notably, we did not see any noticeable difference in the number of nuclear vs cytoplasmic inclusions after CDNF treatment. It is clear that the nature and distribution of mHtt aggregates in different neurons and how these can be influenced by CDNF during the course of HD will require more investigations in the future. However, further development of the detection and quantifying method based on AI for analysis of aggregates in HD models or other neurodegenerative diseases where aggregates are presented is valuable. Moreover, this method could replace classical stereological but more biased and time-consuming methods.

Striatal cells that express mHtt have reduced activity of the ubiquitin–proteasome system (UPS) and, which in turn can increase their vulnerability to ER stress^57^. Previously, it was reported that CDNF is involved in the regulation of ER stress in vitro and in vivo in animal models of PD and ALS^22,24^. It was reported that N171-82Q mice had increased levels of ER stress markers (GRP78, CHOP and XBP1) in the striatum^58^. Additionally, N-terminal mHtt fragments with longer polyQ tract (53Q-120Q) increased ER stress markers and induced cell death in vitro in PC6.3 neuronal cells^59^. It has been reported that there is also an increase in the levels of *GRP78* and *CHOP* mRNA in brain tissues of HD patients^60^. In our study, we observed a tendency towards an increased striatal expression of ER stress markers in N171-82Q mice as compared to WT mice and a lower expression levels of ER stress markers after CDNF treatment as compared to PBS treatment in N171-82Q mice. The most pronounced effect of CDNF was observed with *CHOP* mRNA, however, the data did not reach statistical significance, possibly due to the low number of animals examined. Further experiments are therefore necessary to study ER stress and CDNF effects in these mice in the future using higher number of animals and probably also later endpoints than applied here.

BDNF is a NTF that has been linked to the pathophysiology of HD in different models^18,61^. BDNF is anterogradely transported from cortical neurons to the striatum^62^. We observed here that CDNF significantly increased the BDNF levels in cultured striatal neurons expressing mHtt with 109Q repeats. In contrast, there was no effect of CDNF in control cells expressing normal Htt with 7Q repeats. The underlying reason for this is unclear, but it is known that CDNF particularly influences neurons during cell stress, which is present in mHtt cells^23,43,54,57,59^.

Most interestingly, in the present study, we also noted that the levels of *BDNF* mRNA in the hippocampus were significantly lower in the N171-82Q mice than in WT controls. In contrast, we could not detect alterations in striatal and cortical *BDNF* mRNA levels between groups. Previously, a decrease of *BDNF* mRNA in the hippocampus was also reported in the R6/1 mouse model of HD^63^. The significance of reduced *BDNF* mRNA level in the hippocampus in HD is not clear at the moment, but may reflect an impairment of cognitive functions such as learning and memory. A previous study showed that CDNF is involved in long-term memory consolidation when administrated intrahippocampally in the mouse model of AD^64^. We found that chronic CDNF administration significantly increased the mRNA level of hippocampal *BDNF* in N171-82Q mice as compared to PBS-treated N171-82Q mice. These data demonstrate the possible involvement of CDNF in the protection of cognitive functions of the N171-82Q model of HD, particularly memory formation. To investigate this further behavioral tests are needed to study learning and memory performance after CDNF treatment in different HD mouse models. Moreover, the underlying mechanisms of how CDNF affects *BDNF* expression warrant investigation. It was previously reported that CDNF can induce pro-survival signaling in cells mediated by the IRE1α/XBP1 pathway^23,33^. Moreover, it has been shown that XBP1s can modulate BDNF levels in the hippocampus^65^. This may be linked to our finding of an increased BDNF expression in the hippocampus by CDNF.

Previously some NTFs, such as glial cell line‐derived neurotrophic factor (GDNF) and neurturin (NRTN) have been tested in the N171-82Q mouse model of HD and showed neuroprotective effects. However, in these studies GDNF and NRTN were delivered using AAV-delivery, which can limit future applications in clinical trials^13,14^. The present study employing a chronic intrastriatal CDNF protein administration revealed beneficial effects of CDNF on the behavior and BDNF mRNA expression in the N171-82Q mouse model of HD. A possible influence of CDNF on mHtt inclusions and aggregate species is not clear from this study and will require further studies. Along with this, more studies on the precise molecular and cellular mechanism by which CDNF alleviates neurodegeneration in HD models are also warranted.

## Conclusions

We report here the beneficial effects of a chronic intrastriatal CDNF infusion on motor coordination in the transgenic N171-82Q mouse model of HD. In this study, CDNF did not decrease mHtt aggregates or ER stress markers in the striatum likely because of low amount of protein present. Unexpectedly in our study, we noted an increase in *BDNF* mRNA levels in the hippocampus in CDNF-treated N171-82Q mice as compared with PBS-treated N171-82Q mice, and there was also an upregulation after CDNF of BDNF in striatal neurons expressing mHtt. The present results on the N171-82Q transgenic mouse model together with our previous findings using the HD QA-acid toxin model emphasize the potential of CDNF as a potential drug candidate for the treatment of HD. As evident from this study, the use of a deep learning neural network approach is a useful and time-sparing method to quantify Htt aggregates and likely thereby also other protein inclusions present in other neurodegenerative disorders.

## Supplementary Information

Supplementary Figures.

## Data Availability

The datasets used and/or analyzed during the current study available from the corresponding author on reasonable request.
