# Supplementary material for: Beneficial behavioral effects of chronic cerebral dopamine neurotrophic factor (CDNF) infusion in the N171-82Q transgenic model of Huntington’s disease
Source: Sci Rep. 2023 Feb 20;13:2953. doi: 10.1038/s41598-023-28798-4 (PMC9941578; doi:10.1038/s41598-023-28798-4)
Supplement: Supplementary file 1 — Supplementary Figures. [file 41598_2023_28798_MOESM1_ESM.docx]

**Beneficial behavioral effects of chronic cerebral dopamine neurotrophic factor (CDNF) infusion in the N171-82Q transgenic model of Huntington’s disease**

**Stepanova P^1^, Kumar D^2^, Cavonius K^3,4^, Korpikoski J^1^, Sirjala J^1^, Lindholm D^3,4^, and Voutilainen MH^1^.**

^1^Faculty of Pharmacy, University of Helsinki, Helsinki, Finland

^2^Biomedicum, Aiforia Technologies, Helsinki, Finland

^3^Medicum, Department of Biochemistry and Developmental Biology, Faculty of Medicine, University of Helsinki, Finland

^4^Minerva Foundation Institute for Medical Research, Biomedicum, University of Helsinki, Finland

Supplementary information


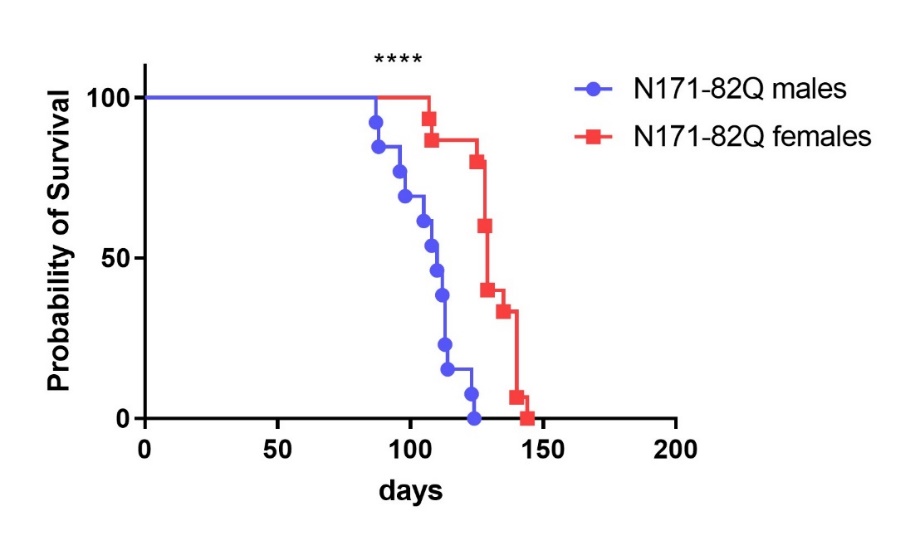


**Supplementary Fig 1. Survival percentages for N171-82Q animals.** N171-82Q males demonstrated a significantly shorter lifespan than females. ****p<0.0001, log-rank Mantel-Cox test. N number per group = 13-15.

**Mouse primers**

NM_007837.4 Mus musculus DNA-damage inducible transcript 3 (Ddit3), transcript variant 1, mRNA

qCHOP_Fwd: 5’ CCAACAGAGGTCACACGCAC

qCHOP_Rev: 5’ TGACTGGAATCTGGAGAGCGA

product length = 53

NM_001081304.1 Mus musculus activating transcription factor 6 (Atf6), mRNA

qATFa_Fwd: GGACGAGGTGGTGTCAGA

qATF6a_Rev: GACAGCTCTTCGCTTTGGAC

product length = 61

NM_001163434.1 Mus musculus heat shock protein 5 (Hspa5), transcript variant 1, mRNA

qBiP_Fwd: 5’ ACCCTTACTCGGGCCAAATT

qBiP_Rev: 5’ AGAGCGGAACAGGTCCATGT

product length = 51

NM_001271730.1 Mus musculus X-box binding protein 1 (Xbp1), transcript variant 2, mRNA

qXBP1s_Fwd: 5’ GAGTCCGCAGCAGGTG

qXBP1s_Rev: 5’ GTGTCAGAGTCCATGGGA

product length = 65

NM_007393.5 Mus musculus actin, beta (Actb), mRNA

qbActin_Fwd: 5’ CTAAGGCCAACCGTGAAAAG

qbActin_Rev: 5’ ACCAGAGGCATACAGGGACA

product length = 104

NM_009716.3 Mus musculus activating transcription factor 4 (Atf4), transcript variant 1, mRNA

qATF4 Fwd: 5’ ATG GCC GGC TAT GGA TGA T

qATF4 Rev: 5’ CGA AGT CAA ACT CTT TCA GAT CCA TT

product length = 113

NR_003280.2 Mus musculus 5.8S ribosomal RNA (Rs5-8s1), ribosomal RNA

q5.8S Fwd: 5’ GCGCTAGCTGCGAGAATTAATGTG

q5.8S Rev: 5’ CAAGTGCGTTCGAAGTGTCGATGA

product length = 66

NM_007540.4 Mus musculus brain derived neurotrophic factor (Bdnf), transcript variant 1, mRNA qBDNF_Fwd: 5’ GGCCCAACGAAGAAAACCAT

qBDNF_Rev: 5’ AGCATCACCCGGGAAGTGT

product length = 55

**
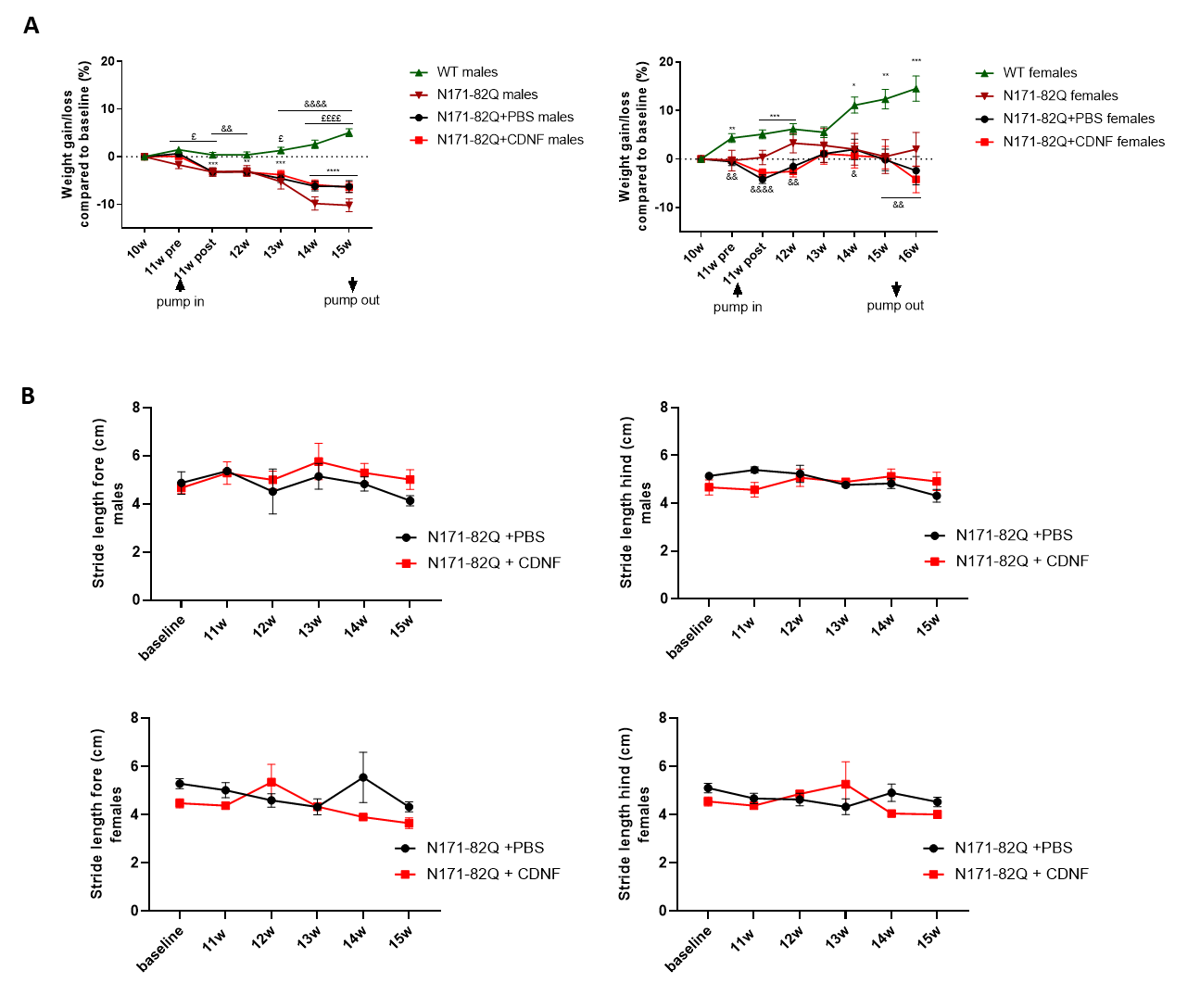
Supplementary Fig 2. Primers for qPCR analysis.**

**Supplementary Fig 3.** (A) Ratio of weight gain to weight loss. CDNF did not have an effect on the weight of N171-82Q mice. *, comparison between WT and N171-82Q+CDNF males; &, comparison between WT and N171-82Q+PBS males; £, comparison between WT and untreated N171-82Q males. ****p<0.0001, ***p<0.001, **p<0.01, *p<0.05, mixed-effect ANOVA test, Tukey post hoc test. N number = 24 (N171-82Q+CDNF), 27 (N171-82Q+PBS), n=13 (WT) and n=8 (N171-82Q groups). *, comparison between WT and N171-82Q+CDNF females; &, comparison between WT and N171-82Q+PBS females. Values are expressed as group mean± SEM. ****p<0.0001, ***p<0.001, **p<0.01, *p<0.05, mixed-effect ANOVA test, Tukey post hoc test. N number = 14 (N171-82Q+CDNF), 19 (N171-82Q+PBS), n=6 (WT and N171-82Q groups). (B) Motor coordination in Digigait test. Stride length on the treadmill. Animals did not show the difference in stride length. Values are means± SEM. N number per group =3-8.


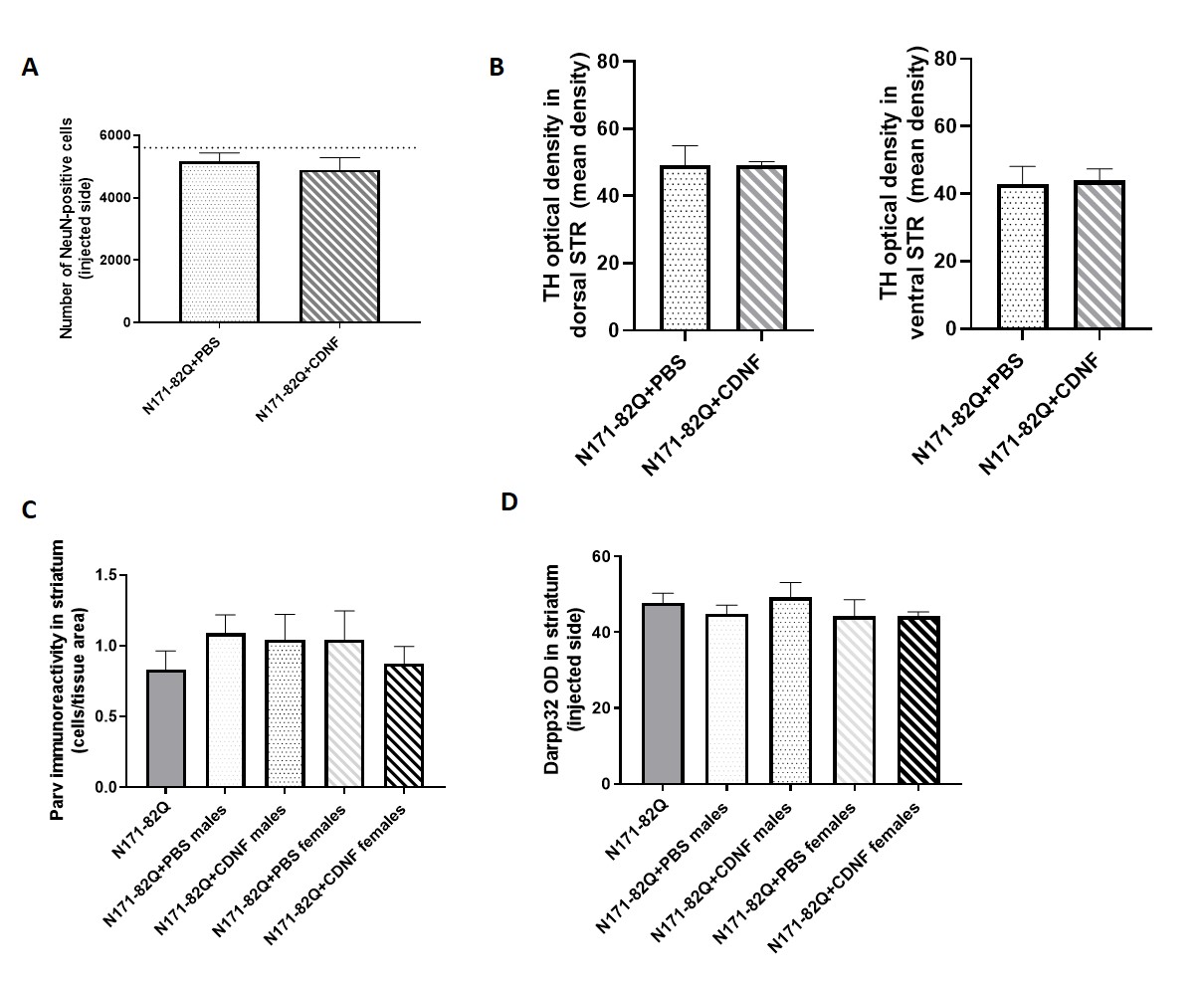


**Supplementary Fig 4. Immunohistochemistry in N171-82Q mice after chronic CDNF administration.** (A) NeuN-Immunohistochemistry. Coronal sections of the striatum show no change in NeuN-immunoreactivity in the striatum of N171-82Q animals. N = 6-9. (B) TH-Immunohistochemistry. No change was found in the number of TH+ fibers in the ventral and dorsal striatum of N171-82Q animals. N = 3-8. (C) Parvalbumin-immunohistochemistry. There was no statistical change in Parv-immunoreactivity in the striatum of N171-82Q mice. N = 4-7. (D) Darpp32-Immunohistochemistry. There is no statistical change in Darpp32-immunoreactivity in N171-82Q. N = 3-11. Immunopositive cells were counted in the 3-5 coronal sections and expressed as mean ± SEM of the total number of cells in the same section.


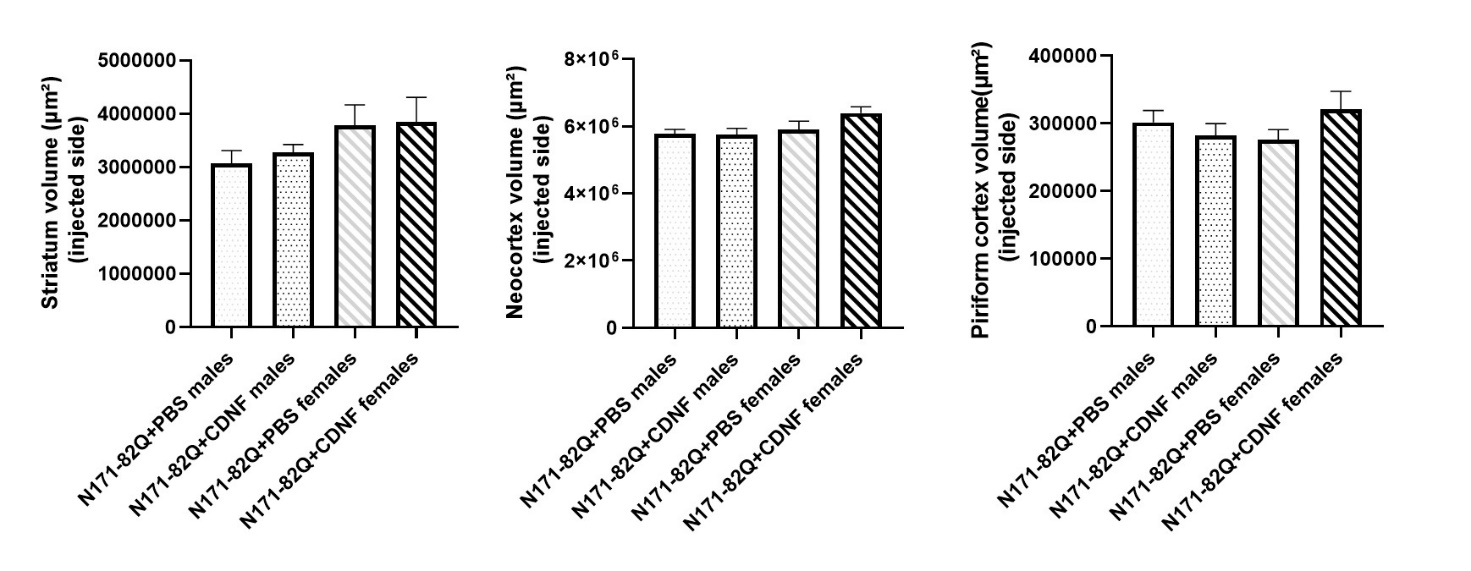
**Supplementary Fig 5. Brain areas volume.** There is no significant difference in the volume of brain areas (striatum, neocortex, piriform cortex) in the two genders. Results expressed as mean ± SEM. N number=5-12.

**
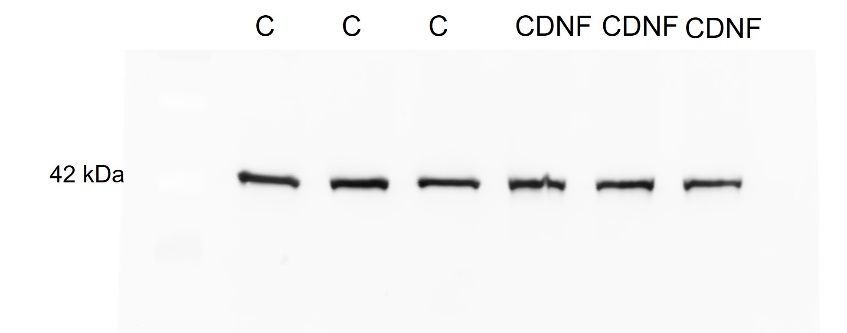

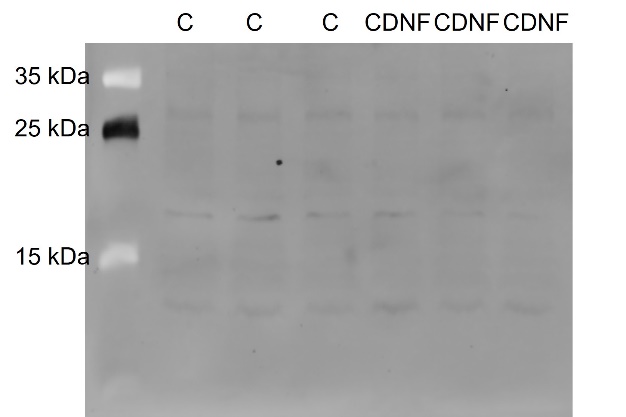
A B**

**Supplementary Fig 6. Original immunoblots for mature BDNF (A) and the GAPDH control (B).** Mutant huntingtin expressing striatal cells were stimulated with PBS (control) or 100ng/ml CDNF for 24h. Left 3 lanes are controls; Right 3 lanes are cells with CDNF. Mature BDNF is shown at 14 kDa, below the 15 kDa protein marker. GAPDH is shown at 42 kDa, above the 35 kDa protein marker. C-control.
